# Quaternary Mixed Oxides of Non-Noble Metals with Enhanced Stability during the Oxygen Evolution Reaction

**DOI:** 10.1021/acsami.4c10234

**Published:** 2024-10-13

**Authors:** Alexis Piñeiro-García, Xiuyu Wu, Esdras J. Canto-Aguilar, Alice Kuzhikandathil, Mouna Rafei, Eduardo Gracia-Espino

**Affiliations:** Department of Physics, Umeå University, SE-901 87 Umeå, Sweden

**Keywords:** mixed oxides, functional oxides, single-rutile phase, oxygen evolution, metal stabilization

## Abstract

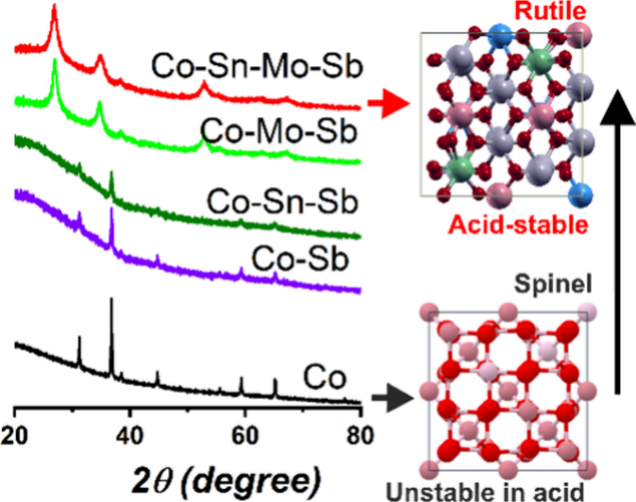

Robust electrocatalysts required to drive the oxygen evolution reaction (OER) during water electrolysis are still a missing component toward the path for sustainable hydrogen production. Here a new family of OER active quaternary mixed-oxides based on X–Sn–Mo–Sb (X = Mn, Fe, Co, or Ni) is reported. These nonstoichiometric mixed oxides form a rutile-type crystal structure with a random atomic motif and diverse oxidation states, leading to the formation of cation vacancies and local disorder. The successful incorporation of all cations into a rutile structure was achieved using oxidizing agents that facilitates the formation of Sb^5+^ required to form the characteristic octahedral coordination in rutile. The mixed oxides exhibit enhanced stability in both acidic and alkaline environments under anodic potentials with no changes in their crystal structure after extensive electrochemical stress. The improved stability of these mixed oxides highlights their potential application as scaffolds to host and stabilize OER active metals.

## Introduction

The production of efficient and stable electrocatalysts for the OER remains the main challenge to achieving a sustainable hydrogen economy. Nowadays, electrocatalysts that are robust enough to resist corrosion under the harsh environments needed in proton exchange membrane (PEM) water electrolyzers are still based on ruthenium and iridium oxides.^1,2^ As is known, their scarcity, high cost, and finite reserves severely restrict the large-scale production of hydrogen via PEM water electrolysis. These limitations have been driving the development of materials that are expected to reduce the usage of noble metals while still retaining a favorable catalytic activity and exhibiting notorious stability under severe conditions.

A relatively successful strategy has been the design of multicomponent oxides that are stable under acidic and anodic conditions, where the addition of Ru/Ir is used to promote oxygen evolution. Among these, the most common oxides are perovskites,^3,4^ pyrochlores,^5,6^ polyoxometalates,^7,8^ and mixed oxides.^1,9^ An interesting family of metal oxides is based on rutile oxides of first-row transition metals (e.g., TiO_2_, VO_2_, CrO_2_, and MnO_2_). These are appealing alternatives as OER electrocatalysts due to their abundance, low cost, and fascinating electronic properties arising from their partially filled d-subshells.^10,11^ This last feature enables tuning of the interaction with OER intermediates to mimic that of noble metals.^12^ Despite these advantages, noble-metal free rutile oxides such as β-MnO_2_ and TiO_2_ still require large overpotentials, typically above 700 mV, to initiate the OER.^13,14^ Nevertheless, the exceptional corrosion resistance seen in these rutile oxides makes them great candidates to host and stabilize other OER active metals.^15,16^ For instance, the group of N. S. Lewis reported the production of mixed oxides based on Ni–Mn–Sb with rutile structure with a stable operation (η_10,OER_ = 735 mV, 1 M H_2_SO_4_) for up to 168 h.^17^ Recently, they also reported a Mn–Sb rutile oxide with excellent durability in HClO_4_.^18^ Similarly, D. G. Nocera et al. reported the formation of rutile Ni–Fe–Pb oxide (Ni:Fe:Pb 1:1:1) with stable OER activity for up to 20 h at pH = 2.^19^ These studies demonstrate that separating stability from activity enables us to address one problem at a time. Given that activity without stability holds little value, it becomes evident that stability should take precedence.

Therefore, in this work, we report the production of a new family of nonstoichiometric quaternary mixed-oxides based on X–Sn–Mo–Sb (X = Mn, Fe, Co, or Ni). These mixed oxides crystallize in the rutile phase and exhibit a randomized atomic motif with diverse oxidation states resulting in the formation of cation vacancies and local disorder. The mixed oxides exhibit enhanced stability in both acidic and alkaline environments under anodic potentials, retaining their crystal structure and cation composition. We observe that the crystal structure plays a fundamental role in enhancing the stability of all of its components. Finally, Co- and Mn-based mixed oxides exhibit significant activity toward the OER, opening new routes to engineer active and stable noble-metal free OER electrocatalysts.

## Experimental Methods

### Materials and Reagents

Tin(IV) chloride pentahydrate (SnCl_4_·5H_2_O, 98%), antimony chloride (SbCl_3_, ≥99%), iron(III) chloride (FeCl_3_, 97%), cobalt(II) chloride hexahydrate (CoCl_2_·6H_2_O), nickel(II) chloride hexahydrate (NiCl_2_·6H_2_O), manganese(II) chloride tetrahydrate (MnCl_2_·4H_2_O), sulfuric acid (H_2_SO_4_, 95–97%), potassium hydroxide (KOH, ≥85%), ammonium fluoride (NH_4_F, 98%), and hydrochloric acid (HCl, 37%) were acquired from Merck (Sigma-Aldrich). Molybdenum (particle size of 170 mesh) and tungsten (size of 170 mesh) powders were acquired from Alfa Aesar. Hydrogen peroxide (H_2_O_2_, 35%), and absolute ethanol (EtOH, >99.9%) were purchased from VWR. All chemicals were used as received. Fluorine-doped tin oxide (FTO) coated glass slides (7 Ω sq^–1^) were acquired from Merck (Sigma-Aldrich).

### Catalysts Production

Quaternary mixed-oxides (MO) based on X–Sn–Mo–Sb (X = Mn, Fe, Co, or Ni) were grown in the form of a coating onto FTO glass substrates using solution precursor plasma spraying (SPPS). First, FTO substrates (1 cm × 2 cm) were thoroughly cleaned using EtOH/H_2_O (70% v/v) in an ultrasonic bath for 1 h and then rinsed with water (18.2 MΩ·cm). In a typical procedure, Mo-peroxo complexes (MoO_4_^2–^) were first produced by dissolving metallic Mo powder in H_2_O_2_ until a metal concentration of 0.4 M was achieved.^20^ Then, different aqueous precursor solutions containing X, Sn, Mo, and Sb with a molar ratio of 1:1:1:2 with a total metal concentration of 0.1 M were used as feedstock for the SPPS process. The solution precursors were prepared by dissolving X, Sn, and Sb salts in 45 mL of EtOH by using an ultrasonic bath. After 5 min, 5 mL of Mo-peroxo complexes were added while still in the sonication bath. Later, 8.3 mL of HCl was poured directly into the solutions followed by the addition of 1.8 mL of NH_4_F (40% w/v). The solutions were left to rest for 10 min under sonication. Finally, diionized (DI) water was added to obtain a total volume of 100 mL; these solutions were used during the SPPS process. The MO coatings were produced using an atmospheric plasma spraying system (Metallisation Ltd., Met-PCC (PLAS), PL50 pistol with a 6 mm nozzle). Argon (50.0 NL min^–1^) was employed to create the plasma while applying 500 A resulting in a power of 25 kW. The solution precursor (15 mL min^–1^) was mixed with N_2_ gas (3 NL min^–1^) and introduced to the plasma plume through a nozzle. The plasma torch was controlled by a robotic arm (ABB 2600) using a raster spraying pattern (250 mm s^–1^ as lateral velocity, 4 mm as vertical displacement while maintaining 150 mm of distance to the substrate) until an area of ∼180 cm^2^ was covered forming one layer. A complete coating was achieved by applying a total of 16 layers. Eight FTO glass substrates were placed in the center of the spraying area where a mask limited the coated surface to 1 × 1 cm^2^ in each substrate. After the spraying process, the coated FTO glass substrates were annealed in air at 500 °C for 2 h (temperature ramp of 1.6 °C min^–1^, total time ∼7 h). The samples were labeled as Mn-MO, Fe-MO, Co-MO, and Ni-MO. Additional samples were prepared on Ti fiber felt (thickness of 0.2 mm) and Ni mesh (diamond-shaped expanded Ni mesh, thickness of 0.76 mm, wire width of 0.88 mm, and a short (long) way of mesh of 1 mm (3 mm)). The synthesis process was kept unchanged. Samples prepared on these substrates were labeled as X-MO@Ti, or X-MO@Ni, respectively.

### Materials Characterization

Scanning electron microscopy (SEM) studies were conducted on a Carl Zeiss Merlin microscope equipped with an energy-dispersive X-ray spectrometer (EDX). X-ray photoelectron spectroscopy (XPS) was performed on a Kratos Axis Ultra DLD electron-spectrometer with a monochromatic X-ray source (Al Kα = 1486.6 eV). X-ray diffraction (XRD) studies were conducted on a PANalytical X’pert diffractometer (λ = 1.5406 Å, Cu Kα) in the range of 20–95° (step size of 0.01313° and 1.175 s per step) at atmospheric conditions. Raman spectra were recorded in a Renishaw Qontor Raman spectrometer using a 532 nm laser diode (calibrated with Si crystal at 521 cm^–1^), operated at 10% of power for 3 s with a total of 3 accumulations.

X-ray absorption spectroscopy (XAS) studies were performed to evaluate the L-edges of Co and Mn and the K-edge of O. The experiments were performed at the FlexPES (Flexible PhotoElectron Spectroscopy) beamline at MAX IV synchrotron source in Lund, Sweden.^21^ The spectra were measured under ultrahigh vacuum conditions at room temperature. An exit slit of 10 μm was used with a photon flux 10^12^ photons s^–1^ and a photon energy resolution of 26 meV. The beam size at the sample was 2 mm × 1 mm (defocused mode). The data were recorded in the surface-sensitive (5–10 Å) partial electron yield (PEY) detection mode, and all spectra were normalized after background subtraction.

### Electrochemical Measurements

The electrochemical measurements were performed in a conventional three-electrode cell at room temperature by using an Autolab electrochemical workstation. The X-MO coatings were used as working electrodes, whereas a Pt wire was used as counter electrode and a Ag/AgCl (3 M KCl) electrode as a reference electrode. All measured potentials were converted to the reversible hydrogen electrode (RHE) using *E*_RHE_ = *E*_cell_ + 0.059 pH + *E*_Ag/AgCl_; *E*_Ag/AgCl_ = 0.210 V vs NHE, with *E*_cell_ being the experimental measured potential. The double layer capacitance was evaluated by cycle voltammetry (CV) in a non-Faradaic region at different scan rates, from 5 to 100 mV s^–1^. The activity of the X-MO electrodes for the OER in acidic ([H_2_SO_4_] = 0.5 M) and alkaline ([KOH] = 1M) conditions was evaluated in the potential window of 1.2–2.2 V vs RHE with a scan rate of 1 mV s^–1^. The stability of the of X-MOs in acid was evaluated by (i) 500 cyclic voltammograms (CVs, 100 mV s^–1^) in the potential window of 1.2–2.0 V vs RHE and (ii) 5000 CVs (100 mV s^–1^, totaling ∼24 h). The stability tests were performed on different sets of samples. In alkaline conditions the stability was evaluated by performing 1000 CVs (100 mV s^–1^) followed by a chronoamperometry test for 24 h. Due to the difference in activity of samples deposited on FTO, a current density of 10 mA cm^–2^ was set for Co-MO and Mn-MO, and 5 mA cm^–2^ was set for Fe-MO and Ni-MO. For samples deposited on Ni mesh (X-MO@Ni), a current density of 10 mA cm^–2^ was used for all electrodes. All the electrolyte solutions were saturated with high purity Ar for 30 min prior any experiment.

### Computational Details

Spin-polarized density functional theory (DFT) computations were performed using the Quantum Espresso code.^22^ The Perdew–Burke–Ernzerhof implementation was used as an exchange–correlation functional. The pseudopotentials were selected from the standard solid-state pseudopotential (SSSP) library optimized for precision 1.2.1.^23^ The systems were geometrically optimized until the components in the atomic forces were less than 5 × 10^–4^ (a.u.), and the total energy change less than 1 × 10^–4^ (a.u.). A kinetic energy cutoff for wave functions of 90 Ry was used, and 1080 Ry was the kinetic energy cutoff for the charge density and potential. Mn-MO and Fe-MO were studied by using 1 × 1 × 2 rutile (*P*4_2_/*mnm*) unit cell (12 atoms total), while for Co-MO and Ni-MO the trirutile unit cell (18 atoms) was used. The spin polarization was initiated with the early transition metals having an apposite spin arrangement resulting in a total magnetization equal to zero. The starting magnetization value for all other atoms was set to zero. The Brillouin zone was sampled using an 8 × 8 × 4 k-grid. Large nonstoichiometric systems were prepared according to the EDX and XPS analysis (discussed in the main text) resulting in nearly cubic cells of ∼19 Å in size containing more than 550 atoms. In this case, the Brillouin zone was only sampled at the γ point, and the threshold for the atomic forces was reduced to 1 × 10^–3^ (a.u.). All other parameters were kept identical.

## Results and Discussion

### Morphology and Chemical Composition of the Mixed Oxides

**Figure 1:**
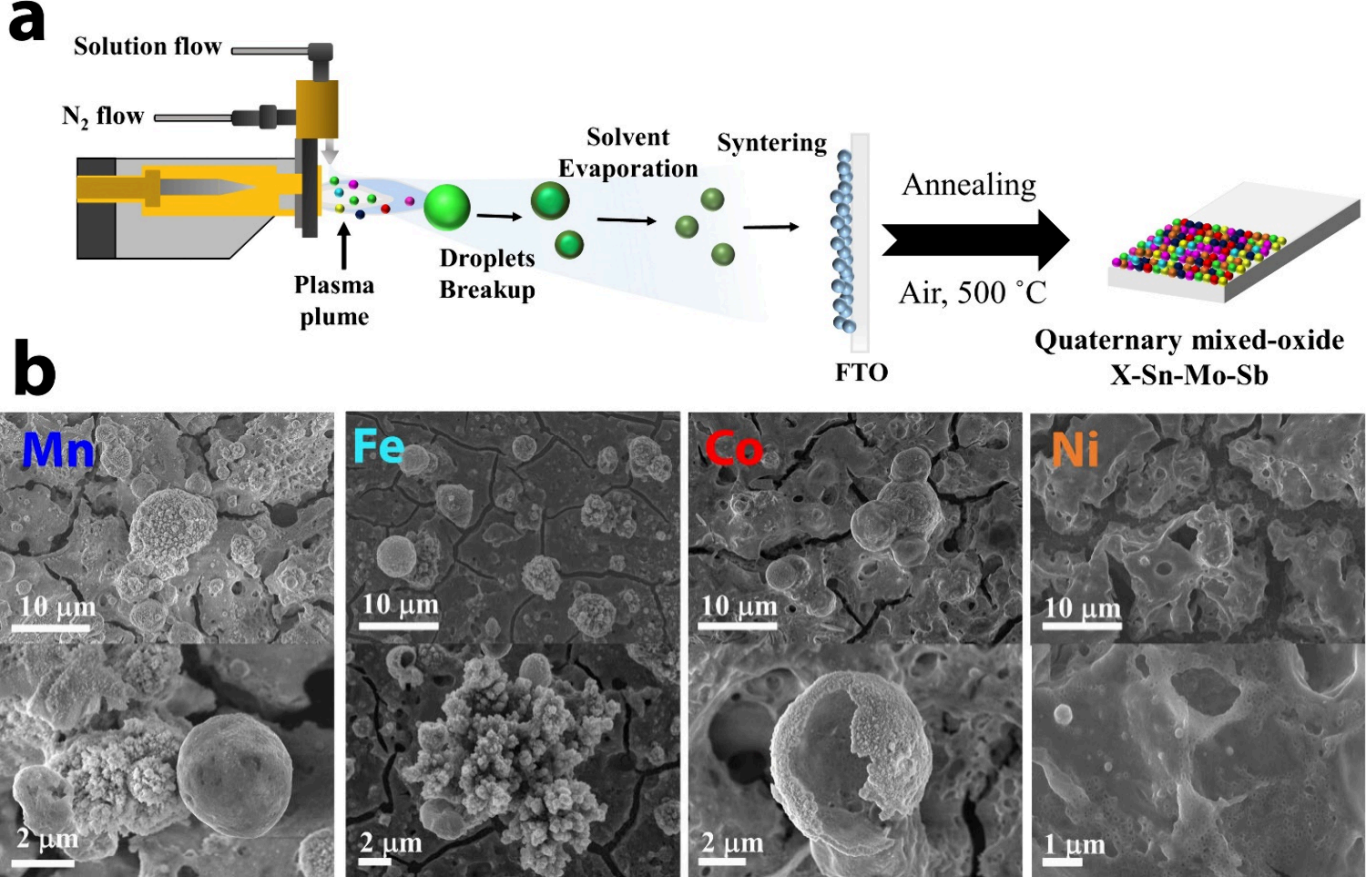
(a) Coatings produced via SPPS using a solution precursor containing the desired cation composition. The mixed oxide coatings are produced by applying several layers followed by thermal annealing in air. (b) SEM images of the quaternary mixed-oxides (X–Sn–Mo–Sb) deposited on FTO, from left to right: Mn-MO, Fe-MO, Co-MO, and Ni-MO.

Quaternary mixed oxides (X–Sn–Mo–Sb, X = Mn, Fe, Co, or Ni) were grown on FTO glass via atmospheric plasma spraying. FTO glass was selected as substrate because of its high stability in acidic and alkaline environments under anodic potentials, which allows investigation in detail of the electrochemical stability and performance of the electrocatalysts under harsh environments. In our SPPS process, illustrated in Figure 1a, the solution precursor is fed into the plasma plume where droplets undergo solvent evaporation, precursor decomposition, particle formation, and sintering (particle agglomeration).^24,25^ As a result, the particles are formed in-flight before reaching the substrate. The coating is then formed by the successive deposition of the nanoparticulate material. The as-deposited films were subsequently annealed (500 °C at 2 h) to form the quaternary X–Sn–Mo–Sb mixed oxide (X-MO, X = Mn, Fe, Co, or Ni). All four X-MO coatings exhibit similar characteristics with slight variations in their morphology, as depicted Figure 1b. For instance, Mn-MO, Fe-MO, and Co-MO exhibit microscopic particles (2–4 μm in diameter) with porous walls and hollow cores through the entire surface which are not seen in Ni-MO. These microparticles are comprised of agglomerated nanoparticles, as discussed later, which is a common characteristic of SPPS coatings.^9,26^ Overall, all X-MO coatings exhibit interconnected cavities, voids, and fissures visible throughout the entire coating, a morphology that is beneficial for electrocatalysis due to its additional exposed surface area.

**Figure 2:**
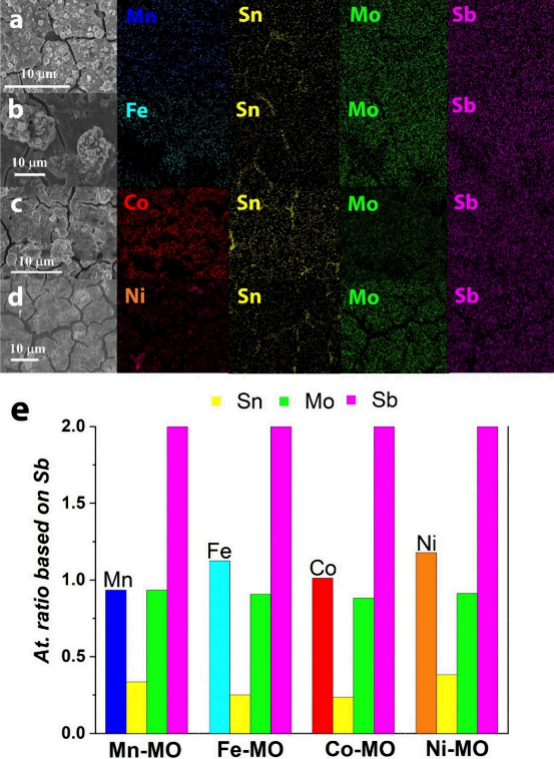
EDX elemental mapping of X-MO coatings produced on FTO: (a) Mn-MO, (b) Fe-MO, (c) Co-MO, and (d) Ni-MO. The Sn contribution might partially originate from the FTO substrate as the regions with high intensity follow the microscopic cracks observed in the SEM images. (e) Metal ratio obtained from EDX normalized with respect to Sb (Sb set to 2). The atomic ratios are 0.93:0.33:0.93:2, 1.12:0.25:0.91:2, 1.01:0.23:0.88:2, and 1.17:0.38:0.91:2 for X:Sn:Mo:Sb, where X = Mn, Fe, Co, or Ni. The precursor solution had an initial metal ratio of 1:1:1:2 for X:Sn:Mo:Sb.

EDX elemental mapping of all X-MO coatings, Figure 2, revealed a homogeneous distribution of all chemical elements without visible signs of agglomeration, apart from Sn. However, the contribution from Sn partially originates from the FTO substrate (which contains Sn) as the regions with high Sn concentration follow the microscopic cracks observed in the SEM images. These cracks are formed after the thermal annealing process (500 °C@2 h) likely caused by differences in thermal expansion coefficients and decomposition of precursor remnants. However, this apparent Sn agglomeration is not observed when using Ti fiber felt as substrate (see Figure S1), indicating that the Sn clustering seen in Figure 2 is indeed due to the FTO substrate. Elemental analysis reveals that the composition of X-MOs deposited on titanium (X-MO@Ti) remains largely unchanged compared to X-MOs deposited on FTO. This indicates that Sn from FTO does not significantly contribute to the overall composition (Table S1). The atomic ratio, reported in Figure 2e, was normalized with respect to (wrt) Sb to facilitate comparison. The content of the early transition metal (Mn, Fe, Co, or Ni), Mo, and Sb was in excellent agreement with the expected metal composition from the precursor solution with an atomic ratio of 1:1:1:2 for X:Sn:Mo:Sb. Note that the contribution of Sn was significantly reduced to less than half of the precursor solution (yellow bars in Figure 2e), even when considering the exposure of the FTO substrate. This phenomenon was also observed when Ti fiber felt was used as a substrate. We found that the Sn content in the deposited material varied depending on the position of the sample within the holder during the SPPS process (Figure S2). We hypothesize that the formation of Sn agglomerates, such as atomic clusters or macromolecules,^27,28^ can lead to the preferential ejection of Sn to the outer regions of the spraying plume, resulting in a reduced Sn deposition on the target area.

### Structure of Mn- and Fe-Mixed Oxides

**Figure 3:**
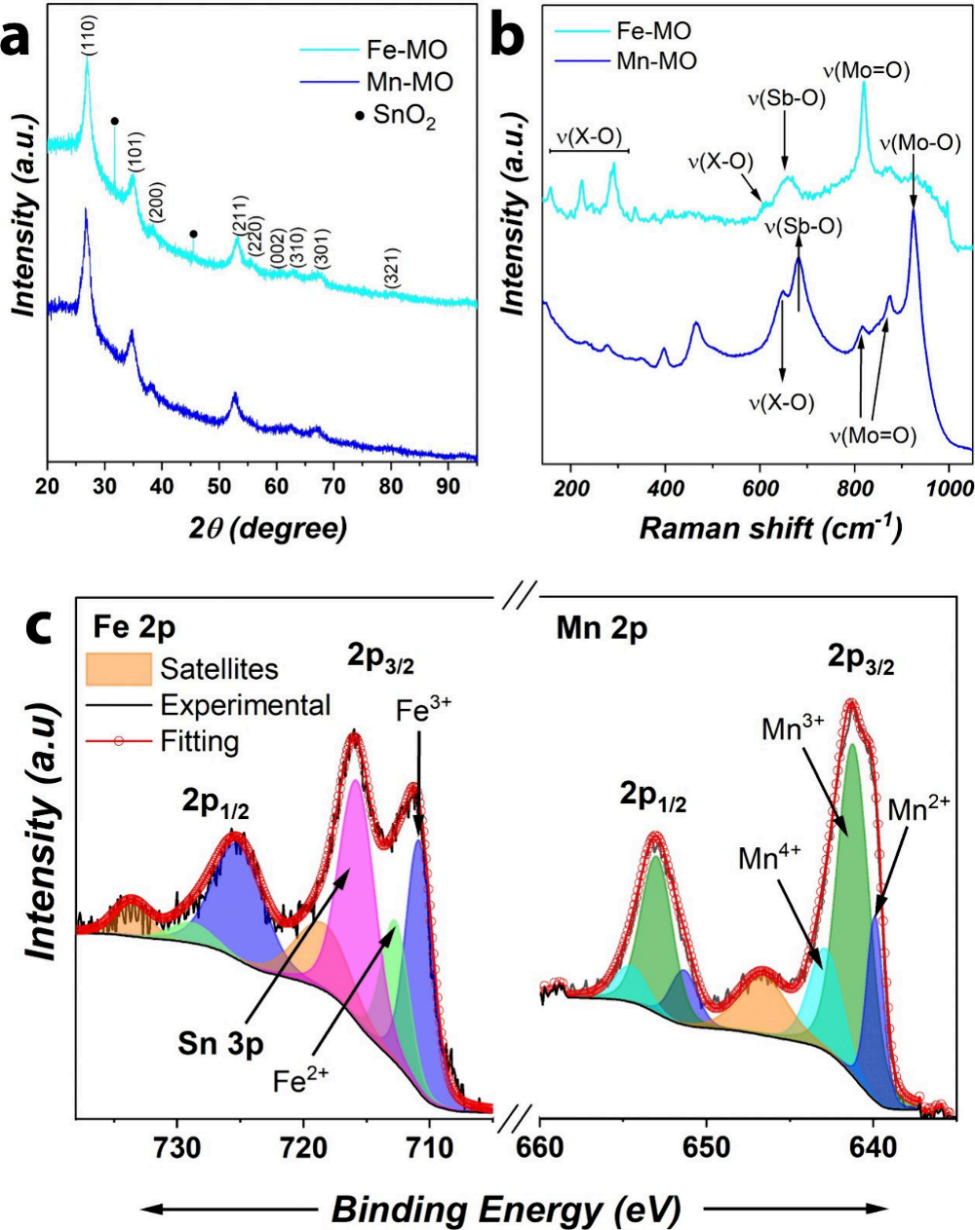
Structural characterization of Mn-MO and Fe-MO produced on FTO: (a) XRD patterns, (b) Raman spectra, and (c) high resolution XPS in the regions of Fe 2p and Mn 2p.

We initiate the discussion with Mn-MO and Fe-MO because of their similarities seen during XRD and Raman studies. XRD patterns in Figure 3a reveal predominant features at 26.7°, 34.8°, and 52.8° for Mn-MO and at 26.9°, 35.1°, and 53.1° for Fe-MO ascribed to the (110), (101), and (211) crystal planes of the tetragonal rutile-type structure with the *P*4_2_/*mnm* space group and empirical chemical formula XSbO_4_ with X = Mn or Fe, in agreement with the ICDD references 01-082-0378 and 00-046-1387, respectively. In this structure, all cations are octahedrally coordinated to oxygen. In addition, there are no features related to Mo, Mn, or Sb oxides (e.g., MoO_2_, MoO_3_, Mn_*y*_O_*x*_, Sb_2_O_*x*_) suggesting a successful incorporation of Fe/Mn, Sn, Mo, and Sb into a single-phase quaternary oxide with a rutile-type crystal structure. However, we noticed that Fe-MO shows a minor segregation of SnO_2_ (features at 31.6° and 45.5° in Figure 3a, ICDD 00-050-1429) which disappeared after the electrochemical stress test, as discussed later, indicating that it was not part of the mixed oxide.

Raman studies were conducted to corroborate the formation of the rutile Mn-MO and Fe-MO. The Raman spectrum of Mn-MO (Fe-MO) in Figure 3b exhibit a band located at 647 cm^–1^ (608 cm^–1^) associated with Mn–O (Fe–O) stretching vibrations corresponding to the A_1g_ mode. This mode appears in the 600–700 cm^–1^ region in oxides with rutile phase such as RuO_2_,^29^ TiO_2_,^30^ MnO_2_,^31^ SnO_2_,^32^ VO_2_,^33^ and Nb_2_O_5._^33^ In addition, both Mn-MO and Fe-MO share a band located at 681 and 660 cm^–1^, respectively, related to Sb–O stretching vibrations also ascribed to the A_1g_ mode. In the case of Fe-MO, the Fe–O stretching vibrations belonging to the E_g_ mode are further seen in the 150–300 cm^–1^ region.^34,35^

Both rutile-type Mn-MO and Fe-MO exhibit additional bands in the 800–1000 cm^–1^ region assigned to Mo–O stretching vibrations. In the case of Mn-MO, additional Raman features at 924 cm^–1^ are associated with Mo–O stretching vibrations, while those seen at 874 and 816 cm^–1^ correspond to the symmetric Mo=O stretching vibrations. However, Fe-MO shows only one band assigned to Mo=O (818 cm^–1^), similar to that of Sb–Mo mixed oxides like Sb_2_MoO_6_ (810 cm^–1^).^36^ The latter suggests that Mo in Fe-MO is likely coordinated to oxygens neighboring Sb atoms. Notably, these Mo–O vibrational modes seen in the rutile mixed oxides are different from those observed in Mo oxides such as MoO_2_ and MoO_3_,^37^ confirming that Mo is indeed part of the mixed oxide rather than forming segregated phases.

The chemical oxidation states of rutile Mn-MO and Fe-MO were investigated by XPS. The survey spectra are depicted in Figure S3. The results are presented in Figure 3c showing the peak fitting of the high-resolution Mn 2p and Fe 2p regions. The Mn 2p region shows a spin–orbital split distance (2p_3/2_–2p_1/2_) of 11.75 eV, which is smaller than the expected for MnO_2_ (11.91 eV),^38^ indicating the absence of this oxide in Mn-MO. This information is in line with the XRD results where no segregated phases were detected. In addition, the 2p_3/2_ region has three peaks at 639.9, 641.2, and 643.0 eV related to Mn^2+^, Mn^3+^, and Mn^4+^, respectively.^39^

On the other hand, Fe 2p region shows features at 710.8 and 712.8 eV corresponding to Fe^3+^ and Fe^2+^ along with a strong satellite at 718.7 eV. Here, the binding energies are shifted to higher energies which discard the existence of Fe oxides.^26^ The Fe 2p region presents an additional peak related to Sn 3p due to the presence of Sn in the quaternary mixed-oxide.^40^ The presence of different oxidation states for Mn and Fe suggests distinct coordination environments in Mn-MO and Fe-MO rutile-type oxides, where distortions and asymmetries in the bonding lengths of the hexacoordinated transition metal ions could lead to changes in the Δ_oct_ as well as splitting of the degenerated e_g_ and t_2g_ orbitals, modifying their electronic configuration and occupation, as observed for other metallic centers in different molecular/crystalline compounds.^41,42^

The XPS spectra of Sn, Mo, and Sb are similar for both rutile-type Mn-MO and Fe-MO. The XPS peak fitting is presented in Figure S4. In the Mo 3d region, the presence of Mo^6+^ is seen by the appearance of the doublet peak 232.3 eV/235.45 eV with a spin–orbit split distance of 3.15 eV,^43^ meaning that Mo is in its highest oxidation state in the quaternary mixed-oxide. For Sn 3d, the peaks at 486.5 and 494.9 eV with a spin–orbit split distance of 8.4 eV confirm the existence of Sn^4+^.^44^ The Sb 3d and O 1s deconvolutions reveal peaks at 530.6 and 540.0 eV with a spin–orbit split distance of 9.4 eV, which correlates well with Sb^5+^.^45,46^ Finally, the O 1s region shows only peaks related to oxygen lattice (O_lattice_, 530.4 eV) and adsorbed water molecules (O_ads_, 531.5 eV).^47^ A summary of both EDX and XPS results is given in Table 1.

**Table 1:** Chemical Formulas and Atomic Ratios of Mixed Oxides Produced on FTO^a^

Mn-MO (Mn_1.12_Mo_0.88_Sn_0.24_Sb_2_O_*x*_)		Fe-MO (Fe_0.92_Mo_0.88_Sn_0.34_Sb_2_O_*x*_)		Co-MO (Co_1.0_Mo_0.88_Sn_0.24_Sb_2_O_*x*_)		Ni-MO (Ni_1.16_Mo_0.88_Sn_0.38_Sb_2_O_*x*_)	
Mn/Sb	0.56	Fe/Sb	0.46	Co/Sb	0.50	Ni/Sb	0.58
Mn/Mo	1.27	Fe/Mo	1.04	Co/Mo	1.13	Ni/Mo	1.31
Mn/Sn	4.66	Fe/Sn	2.70	Co/Sn	4.16	Ni/Sn	3.05
Mo/Sb	0.44	Mo/Sb	0.44	Mo/Sb	0.44	Mo/Sb	0.44
Mo/Sn	3.67	Mo/Sn	2.58	Mo/Sn	3.67	Mo/Sn	2.31
Sn/Sb	0.12	Sn/Sb	0.17	Sn/Sb	0.12	Sn/Sb	0.19
Mn^2+^/Mn^3+^	0.30	Fe^2+^/Fe^3+^	1.71	Co^2+^/Co^3+^	0.68	Ni^2+^/Ni^3+^	3.05
Mn^3+^/Mn^4+^	3.13						

a Atomic ratios are derived from EDX elemental analysis. Ratios of similar cations but different oxidation states were obtained from XPS analysis.

### Structure of Co- and Ni-Mixed Oxides

**Figure 4:**
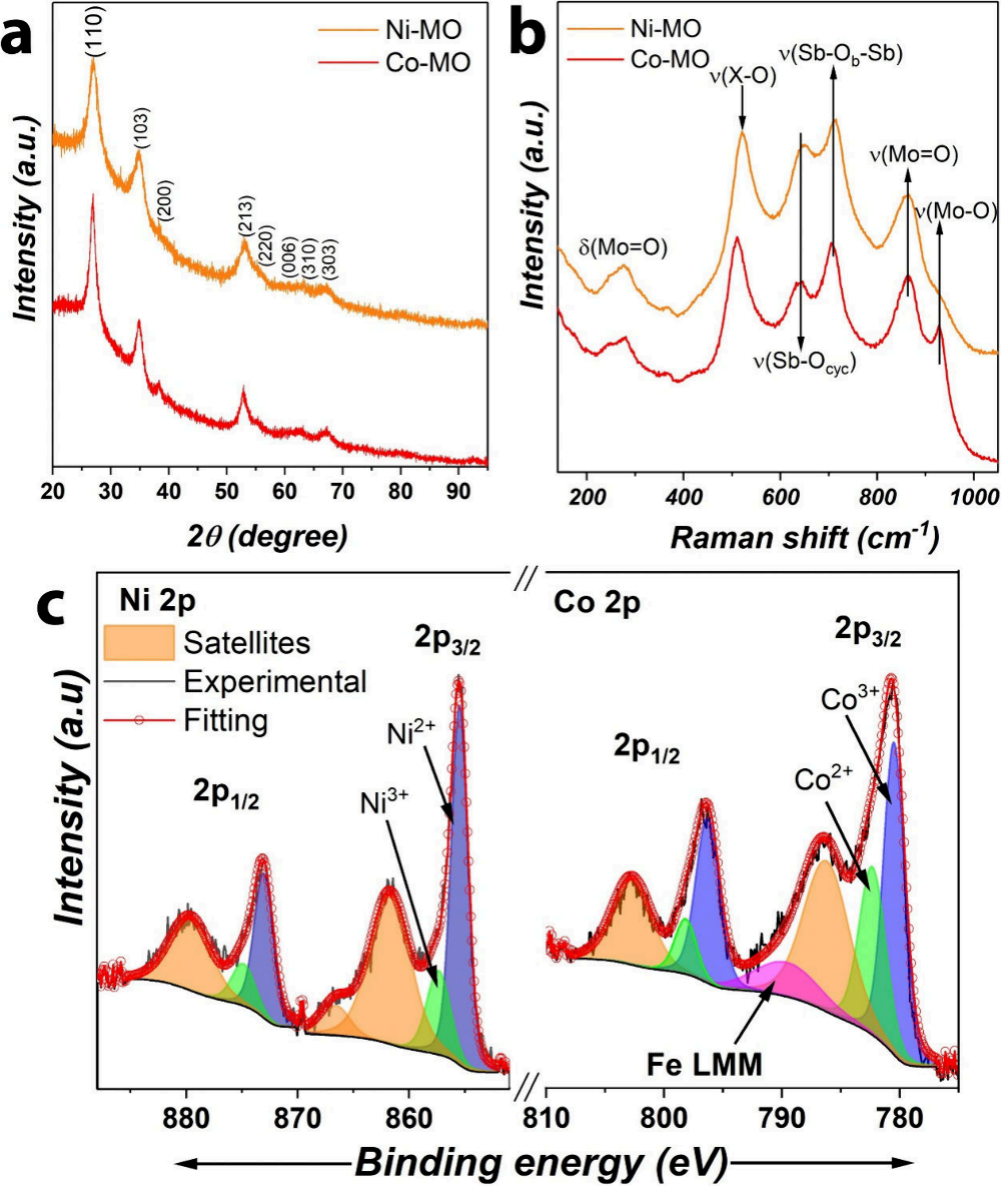
Structural characterization of Co-MO and Ni-MO deposited on FTO: (a) XRD patterns and (b) Raman spectra. (c) High resolution XPS in the regions Ni 2p and Co 2p.

In this section we continue the discussion of Co-MO and Ni-MO. Both quaternary mixed oxides exhibit similar XRD features (Figure 4a) around 27.4°, 35.3°, and 53.3°, associated with the (110), (103), and (213) crystal planes of a trirutile oxide with the *P*4_2_/*mnm* space group and XSb_2_O_6_ (X = Co or Ni) as empirical chemical formula, in good agreement with the diffraction patterns ICDD 00-018-0403 and 00-038-1083, respectively. The trirutile structure is an ordered superstructure of rutile along the c-direction. As previously observed for the Mn-MO and Fe-MO, no evidence of segregated Mo, Sn, or Sb oxides was detected in Co-MO and Ni-MO coatings, confirming that all four distinct cations form a single-phase quaternary mixed-oxide.

The Raman spectra of Co-MO and Ni-MO, Figure 4b, show three main bands in the region 500–700 cm^–1^ assigned to the A_1g_ Raman modes of trirutile structures.^48,49^ The band located at 509 cm^–1^ (522 cm^–1^) corresponds to the Co–O (Ni–O) stretching vibrations. Trirutile structures have a typical formula AB_2_O_6_, B being the atoms that are used as bridging cations or B–O_cycl_ cycles. Thus, the bands in the region 600–800 cm^–1^ are assigned to either Sb–O_b_–Sb vibrations (bridging bonds) or Sb–O_cycl_ (Sb–O forming a ring),^50^ where Sb is coordinated in contiguous octahedral units (Sb_2_O_10_). This behavior is consistent with rutile oxides containing, for example, tantalum (B = Ta).^51,52^ Note that these vibrations are red-shifted with respect to common XSb_2_O_6_,^48,49^ mainly due to the bond stretching frequency that is inversely proportional to the bond length.^52^ This information indicates that Sb could have been replaced by Sn and Mo atoms since no other crystal phases were seen in the XRD patterns (Figure 4b). Refinements in the structure of the materials were obtained after analyzing the region between 800 and 1000 cm^–1^ in the Raman spectrum. We noticed that both Ni-MO and Co-MO exhibit one main band located at ∼863 cm^–1^ related to symmetric O–Mo–O stretching vibrations. Similar to Mn-MO and Fe-MO, Mo might also be replacing or coordinating Sb atoms.^36^ It should be noticed that SnO_2_ was not observed in XRD or Raman studies neglecting possible phase segregation.^27,28^ However, the presence of Sn was evident in EDX studies (Figure 3) and XPS results as discussed below.

The chemical oxidation states of rutile Co-MO and Ni-MO were studied by XPS. Their respective survey XPS spectra are depicted in Figure S3, and the high resolution XPS spectra of Ni 2p and Co 2p are depicted in Figure 4c. The Co 2p_3/2_ region was fitted into two main peaks at 780.6 and 782.44 eV assigned to Co^3+^ and Co^2+^, and a strong satellite at 786.4 eV.^45^ A spin–orbit coupling separation (2p_1/2_–2p_3/2_) of 15.7 eV indicates a predominant existence of Co^2+^ in the rutile structure, as compared to the pure Co^2+^ with an energy split distance of 16 eV.^53^ In addition, we identified a slight contribution of the Fe LMM Auger peak,^54^ a possible impurity that disappeared upon electrochemical stress (discussed later), suggesting that it was not part of the mixed oxide.

The fitting in the Ni 2p_3/2_ region for the Ni-MO coating showed two peaks at 855.5 and 857.4 eV associated with Ni^2+^ and Ni^3+^ cations, respectively. In this case, there is a major contribution of Ni^2+^ as seen by the Ni^2+^/Ni^3+^ ratio of ∼3.0. And thus, the two shakeup satellites at 861.6 and 866.7 eV arise due to large content of Ni^2+^.^55^ Furthermore, the Ni 2p_3/2_ peak is shifted toward higher energies as compared to pure nickel antimonate (NiSb_2_O_6_) with trirutile structure,^46^ indicating either a distortion of the NiO_6_ octahedron^56^ or the presence of vacancies,^55^ which could lead to both 2^+^/3^+^ oxidation states in the transition metal centers, as discussed previously for both Mn-MO and Fe-MO coatings.

The Sn 3d, Mo 3d, and Sb 3d regions in both Co-MO and Ni-MO were fitted following the same procedure used for Mn-MO and Fe-MO, Figure S4, revealing the existence of Mo, Sn, and Sb in their highest oxidation states. Furthermore, the O 1s region shows solely peaks related to oxygen lattice (O_lattice_, 530.4 eV) and adsorbed water molecules (O_ads_, 531.6 eV).^47^ EDX and XPS results are listed in Table 1.

### Formation of the Rutile Oxides

**Figure 5:**
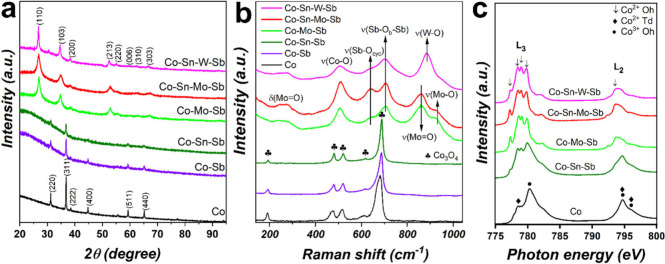
Evolution of the crystal structure of Co-MO produced on FTO: (a) XRD patterns, (b) Raman spectra, and (c) NEXAFS spectra of Co L_2,3_ edges.

The role of the metal cations in the formation of the single-phase mixed oxides is investigated by preparing coatings with an increased number of metals in their composition. We selected Mn-MO and Co-MO as representative of each rutile family. Here we focus on Co-MO with the following increased composition: (i) Co, (ii) Co–Sb (1:2), (iii) Co–Sn–Sb (1:1:2), and (iv) Co–Mo–Sb (1:1:2). All of them were prepared with identical experimental conditions as the quaternary mixed oxide. The results for Mn-MO with similar conclusions are presented in Figure S5. The formation of the rutile structure was monitored by XRD and Raman spectroscopy, and the results are shown in Figure 5. With our current SPPS method, we noticed that the rutile phase was not formed when the precursor solution solely contains Co, Co–Sb, or Co–Sn–Sb. The coating containing only cobalt as a precursor leads to Co_3_O_4_ in the spinel structure which is characterized by having eight tetrahedral and four octahedral sites per cubic unit cell (ICDD 01-080-1533). Interestingly, similar diffraction features are observed for the Co–Sb and Co–Sn–Sb systems (Figure 5a) which can be successfully indexed using the same spinel crystalline phase of the Co_3_O_4_, without evidence of segregated Sb or Sn compounds.^57^ Raman studies (Figure 5b) also support the presence of the spinel structure with bands associated with Co_3_O_4_-like structures (682 cm^–1^, 615 cm^–1^, 516 cm^–1^, and 475 cm^–1^).^58^

On the other hand, the incorporation of Mo in Co–Mo–Sb resulted in the successful formation of the rutile phase. Both Raman spectra and XRD patterns in Figure 5 display similar features to that of the quaternary mixed-oxide (Co–Sn–Mo–Sb) despite not having Sn in the mixture. This indicates that Mo plays a key role during the formation of the rutile phase when using SPPS as the synthesis method. Here, we hypothesize that either the Mo-peroxo complexes (MoO_4_^2–^) or the excess of H_2_O_2_ are acting as oxidizing agents, facilitating the conversion of Sb^3+^ to Sb^5+^. The latter is crucial for the construction of the rutile structure, which consists solely of octahedral sites that require higher oxidation states, in contrast to the spinel structure. We confirmed this hypothesis by replacing the Mo-peroxo complexes by tungsten-peroxo complexes (WO_4_^2–^) in the solution precursor to produce a Co–Sn–W–Sb mixed oxide (Figure 5, magenta line) with rutile structure, as evidenced by XRD. Raman spectroscopy also revealed features (507 cm^–1^, 632 cm^–1^, and 702 cm^–1^) in line with a rutile-type structure.^49,50^ In the new W-based oxide, the strong band centered at 886 cm^–1^ is related to W–O stretching mode, analogue to Sb_2_WO_6_.^59^

As stated before, a similar set of experiments for Mn-MO (Figure S5) led to conclusions similar to those obtained in Co-MO. The single-phase rutile structure is solely formed upon Mo or W-peroxo complexes addition.

We also performed X-ray absorption near edge structure (NEXAFS) measurements at the Co L_2,3_-edge which involves 2p → 3d transitions sensitive to the charge and spin states. The Co L_2,3_-edge spectra of Co-MO is depicted in Figure 5c. The coating containing only Co exhibits a spectrum (main L_3_ 780.2 eV) corresponding to a mixture of Co^2+^ and Co^3+^ with tetrahedral (*T*_*d*_) and octahedral (*O_h_*) coordination, respectively.^60^ This is a clear characteristic of spinel Co_3_O_4_, in agreement with XRD and Raman studies. The Co–Sn–Sb coating develops a gradual energy shift of the main L_3_-edge to 779.0 eV with the emergence of multiple features (marked with arrows) corresponding to Co^2+^ with octahedral coordination (see also Figure S6a).^60,61^ The appearance of Co^2+^ (*O_h_*) features and the reduced intensity of XRD peaks corresponding to the spinel structure (Figure 5a) indicate a reduction in crystallinity and a likely formation of an amorphous Co–Sn–Sb oxide hosting Co^2+^ (*O_h_*).

However, the use of Mo (Co–Mo–Sb coatings) results in better define Co^2+^ (*O_h_*) characteristics, clearly seen by the increase intensity of the lowest energy peak at 777.4 eV. In addition, XRD studies already show the formation of the rutile structure, indicating that the presence of Mo benefits the crystallization of Co-MO into rutile, in which all cations exhibit an octahedral coordination. The Co L_2,3_-edge spectra of both quaternary mixtures, Co–Sn–Mo–Sb and Co–Sn–W–Sb, are similar to Co–Mo–Sb oxide (Figure S6b,c). However, the presence of W results in less define peaks at low energies, likely caused by larger variations in symmetry due to the cation size or the presence of oxygen vacancies.^60^

The relative peak intensity *I*(L_3_)/*I*(L_2_) and the branching ratio *I*(L_3_)/(*I*(L_3_) + *I*(L_2_)) was evaluated by identifying the maximum intensity of the normalized L_3_ and L_2_ peaks. We observed that the relative peak intensity increases from 1.44 for the unary oxide Co_3_O_4_ to 2.47 for quaternary oxides (Table S2) revealing an increase in electron occupancy in the d-orbitals of Co. This indicates a top-surface (5–10 Å) dominated by Co^2+^, which is in contrast to the results from XPS (Table 1) where a larger contribution from Co^3+^ is observed. This discrepancy could be a result of the different penetration depths between XPS (up to 10 nm) and NEXAFS in PEY mode (up to 10 Å). On the other hand, the branching ratio increased from 0.59 to 0.72 indicating the presence of a high spin state of Co^2+^ (*O_h_*).^61^ The presence of high spin Co^2+^ has been correlated to an enhanced activity and stability in various electrochemical processes.^62,63^

To further corroborate the viability of producing Co-MO with a rutile structure, we performed theoretical simulations using density functional theory. We first evaluated the feasibility of introducing Mo and Sn into the analogous rutile oxide CoSb_2_O_6_ which resembles our Co-MO as seen by XRD. A series of simulations in which Co and Sb were replaced by either Mo or Sn, individually or combined (e.g., 4Mo + 2Sn), revealed that both Mo and Sn have no preferred crystallographic site, indicating that they could occupy any of the octahedral sites forming the rutile structure. We only observed differences in energy of less than 1 meV per atom when replacing the same cation (either Co or Sb) at different crystallographic sites; see Figure S7a,b and Table S3 for further details. Interestingly, all the constructed CoMoSnSbO_*x*_ crystals exhibited a favorable formation energy irrespective of the atomic configuration and number of replaced atoms. Therefore, these results are in good agreement with the formation of a quaternary mixed-oxide containing Co, Mo, Sn, and Sb with a disordered-rutile structure instead of the ordered-trirutile structure of XSb_2_O_6_ oxides. In addition, the simulations suggest that the atomic motif of the cations is likely random (i.e., not occupying a specific site in the lattice); such a configuration is seen on disordered rutile oxides such as FeSbO_4_ or MnSbO_4_.

**Figure 6:**
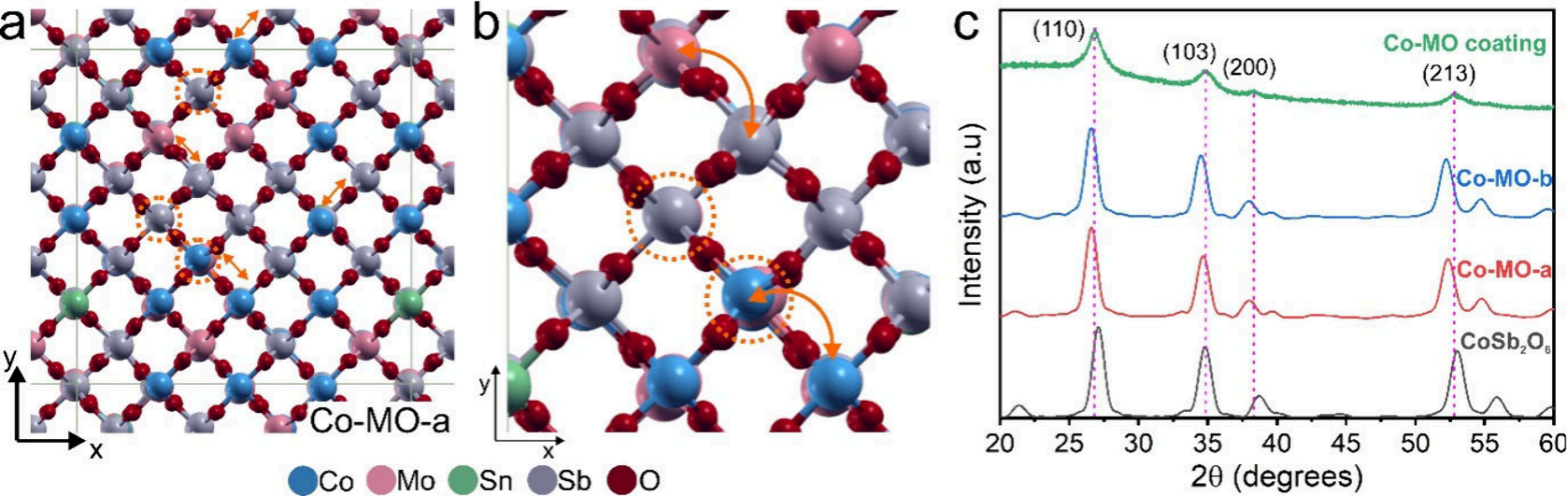
(a) Geometrically optimized atomic model of simulated quaternary mixed-oxide Co-MO-a. Their elemental composition is based on EDX and XPS analysis of Co-MO (CoMo_0.88_Sn_0.24_Sb_2_O_*x*_). The cation occupation in the lattice was randomly assigned. The straight gray lines represent the simulation cell. Dashed circles indicate cation vacancies. The arrows indicate elongated metal–oxygen–metal bonds. (b) A close-up view near vacancies. (c) Comparison of the experimental XRD pattern of the Co-MO coating with the simulated XRD patterns of three nanocrystals with a rutile structure: CoSb_2_O_6_, Co-MO-a, and Co-MO-b.

With this in mind, we constructed two larger atomic models of Co-MO by replicating the trirutile unit cell (4 × 4 × 2) of CoSb_2_O_6_. The resulting Co-MO atomic model was a nearly cubic supercell of ∼19 Å in size and more than 550 atoms. Co and Sb were randomly replaced by Mo and Sn according to the elemental composition and cations oxidation states obtained from EDX and XPS stated in Table 1. The final cation content is listed in Table S4. The total number of cations was adjusted to maintain charge neutrality by considering their diverse oxidation states. The latter resulted in crystals with cation vacancies due to the presence of cations with higher oxidation states such as Mo^6+^ and Co^3+^. The atomic models are labeled Co-MO-a and Co-MO-b, and their geometrically optimized structures are shown in Figure 6a and Figure S7c, respectively.

The simulations reveal that the lattice parameter (*a̅* = 4.7340 Å, *c̅* = 9.2850 Å) had only a slight increase when compared to that of pristine CoSb_2_O_6_ (*a* = 4.6539, *c* = 9.2830 Å). This increase is substantially smaller than the one obtained when using smaller simulation cells (Table S3) indicating that the random nature of the atomic motif helps to alleviate preferential distortion that might be caused by the clustering of cations. However, there are clear local distortions caused by a combination of differences in atomic radii (e.g., Mo) and the presence of cavities left by the cation vacancies. The latter is usually the main caused for distortions because some oxygen atoms move closer to their cation companions affecting the octahedral coordination, marked by an arrow in Figure 6a,b. The XRD patterns were simulated by using Debye’s scattering equation to investigate if the observed structural changes had a major impact in the crystal structure relative to rutile CoSb_2_O_6_. The experimental XRD pattern of the Co-MO coating is also included as a comparison. As we can see in Figure 6c, the simulated XRD of Co-MO-a and Co-MO-b shows similar diffraction features to that of CoSb_2_O_6_, but these are slightly shifted toward lower angles indicating increased interplanar distances, in agreement with the experimental observations. The latter is due to the presence of vacancies, changes in chemical composition, and lattice distortion. We also observed that the simulated XRD results exhibit a larger peak shift when compared to the experimental Co-MO pattern. This could suggest that there are other factors that affect its crystal structure not considered in this model. Although these results were obtained with Co-MO, it is likely that similar conclusions can be drawn for Mn-MO, Fe-MO, and Ni-MO, indicating that formation of quaternary mixed-oxides with rutile structure is feasible, as already evidenced by XRD, Raman, and NEXAFS spectroscopy.

### Activity and Stability under Acidic and Anodic Potentials

**Figure 7:**
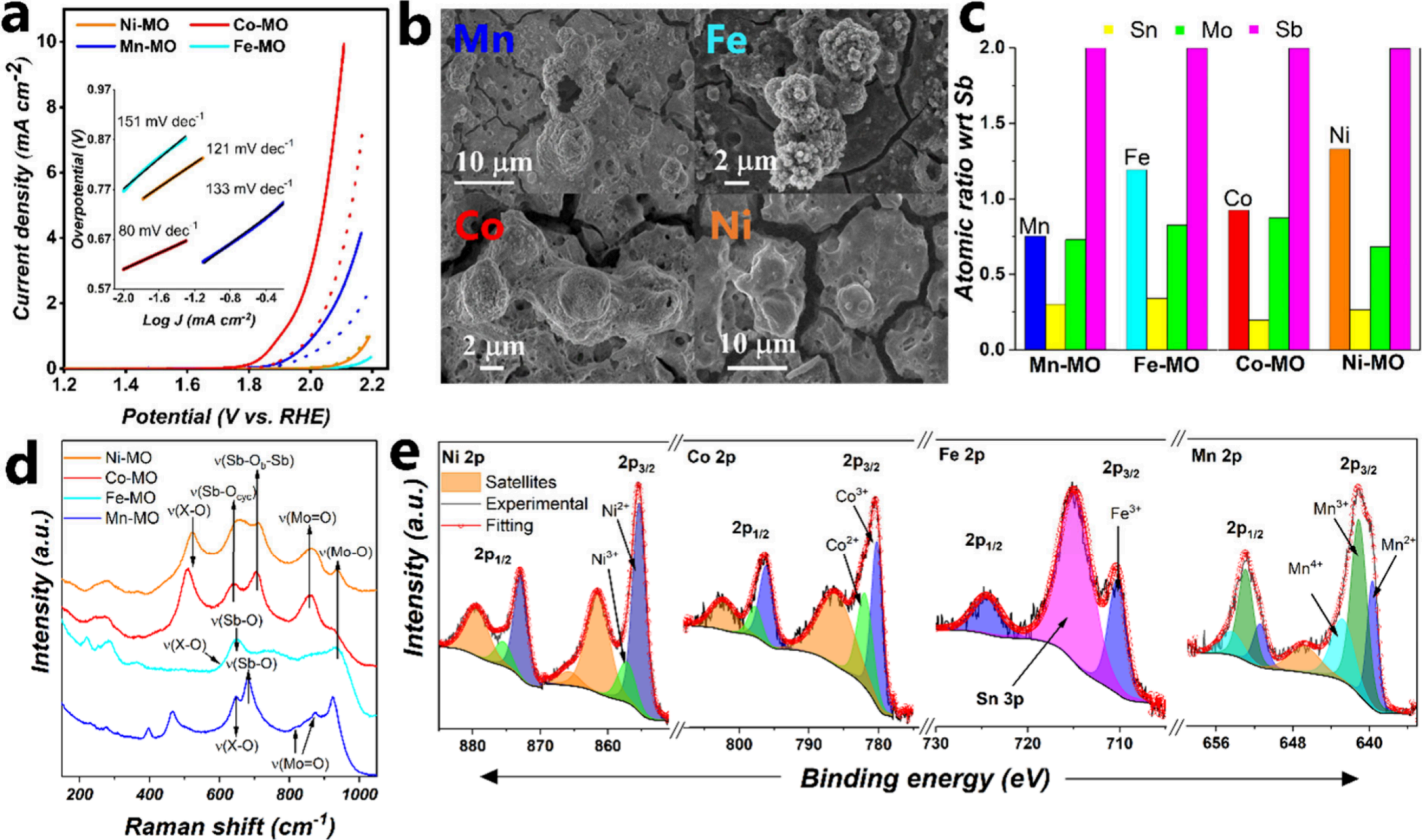
(a) *iR*-corrected polarization curves of the X-MO coatings produced on FTO in 0.5 M H_2_SO_4_ with a scan rate of 1 mV s^–1^ (dash lines correspond to the activity after 500 CVs). The inset shows the Tafel slopes. (b) SEM images. (c) Metal content normalized with respect to Sb. The atomic ratios are 0.75:0.30:0.72:2, 1.19:0.34:0.82:2, 0.92:0.19:0.87:2, and 1.33:0.26:0.68:2 for X:Sn:Mo:Sb, X = Mn, Fe, Co, or Ni. (d) Raman spectra. (e) High resolution XPS in the regions Ni 2p, Co 2p, Fe 2p, and Mn 2p (from left to right), all of them taken after 500 CVs.

We explored the feasibility to employ the X-MO (X = Mn, Fe, Co, Ni) coatings as catalyst to trigger the OER in acid media (pH 0), Figure 7a, in the potential window 1.2–2.2 V vs RHE. Among the catalysts studied, Co-MO and Mn-MO presented the best OER activity in acidic media, followed by Ni-MO and Fe-MO. Co-MO exhibited overpotentials to reach 5 mA cm^–2^ (η_5_) and 10 mA cm^–2^ (η_10_) of 816 mV and 870 mV, respectively, while Mn-MO required 940 mV at η_5_. The Tafel slopes, inset of Figure 7a, have the following trend: Fe-MO (151 mV dec^–1^) > Mn-MO (133 mV dec^–1^) > Ni-MO (121 mV dec^–1^) > Co-MO (80 mV dec^–1^). These values coincide with the water adsorption toward the active site as the rate-determining step (rds), as illustrated in eq 1:^9^
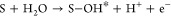
1

In eq 1, S and S–OH* stand for an active site and an intermediary species, respectively. The observed performance is comparable with other mixed oxides (Table S5), while IrO_2_ is reported to have η_10_ in the range of 290–315 mV and a Tafel slope of 58–75 mV dec^–1^.^3,4^ Electrochemical impedance spectroscopy (EIS) measurements (Figure S8) showed similar ohmic resistance (*R*_Ω_) for all X-MOs, but the charge transfer resistance (*R*_ct_) varied. Similar *R*_Ω_ indicates that *R*_ct_ values obtained at 2.2 V are mainly dominated by reaction kinetics and not by a polarization overpotential. Here Co-MO and Mn-MO exhibited the smallest *R*_ct_ (see Table S6) indicating better electrochemical activity for the OER reaction. Although the quaternary mixed-oxides presented high onset for the OER, there are several strategies to improve the activity such as hosting noble metals,^64^ engineering defects,^65^ or increasing the surface area.^66^

Samples sprayed on Ti fiber felt (X-MO@Ti) exhibited improved maximum current density, reduced overpotential, and reduced charge transfer resistance, likely attributed to enhanced conductivity and larger exposed surface area of the Ti fiber felt. However, the Tafel slopes for X-MO@Ti coatings are generally higher than those observed on FTO-based coatings (Figure S9a–d, Table S7). This discrepancy may be due to unaccounted side reactions occurring at the Ti substrate, complicating the accurate determination of the Tafel slope. Electrochemical active surface area (ECSA) measurements (Figure S10 and Table S8) show that X-MO@Ti coatings have comparable values, suggesting that the superior performance of Co-MO is primarily due to its higher specific activity compared to Mn-MO, Fe-MO, and Ni-MO.

The stability of the X-MO coatings in acidic media was initially assessed by performing a series of 500 CVs (scan rate of 100 mV s^–1^) in the potential window of 1.2–2.0 V vs RHE (Figure S11, Figure S9e–h). SEM studies reveal negligible morphological changes after the stress test (Figure 7b) compared to pristine X-MO samples (see Figure 1). Elemental mapping analysis (Figure S12) still reveals a homogeneous metal distribution over the entire coating. The atomic ratio normalized with respect to Sb shown in Figure 7c reveals that the X:Sb (X = Mn, Fe, Co, and Ni) ratio after the electrochemical test is nearly identical to the pristine ones; see also Table S9. It is noteworthy to highlight that minimum to no degradation was observed for Mo, despite the fact that Mo corrosion typically occurs at 0.5 V vs RHE at pH = 0.^67^ Therefore, these results confirm that Mo is well integrated and stabilized into the crystal structure of the quaternary mixed oxide, which in turn boosts its stability in acidic environments.

Raman spectroscopy (Figure 7d) also provided results confirming minor signs of degradation with a slight broadening in the Raman bands suggesting a reduced particle size or particle amorphization.^68^ In addition, the XRD patterns (Figure S13) still reveal features corresponding to a rutile-type crystal structure, in line with the Raman results. Furthermore, the SnO_2_ features observed by XRD in pristine Fe-MO (see Figure 3a) are no longer visible, confirming that the partially segregated Sn was not part of the mixed oxide structure.

The oxidation states of the X-MO coatings were followed by XPS after electrochemical stress, Figure 7e. We observed that Mn-MO still exhibited a similar spin–orbit split distance (11.80 eV) along with features associated with rutile Mn-MO (639.7 eV Mn^2+^, 641.1 eV Mn^3+^, and 642.9 eV Mn^4+^). Fe-MO exhibited solely the peak related to Fe^3+^ (710.2 eV). This result might indicate a possible Fe corrosion within the first few nanometers at the top surface where XPS can penetrate. Nonetheless, EDX analysis, which provides a more in-depth analysis, reveals minimum Fe corrosion, indicating an enhanced stabilization in the rutile-type structure. In the case of Co-MO, the peaks associated with Co^2+^ and Co^3+^ were detected, the main difference being the absence of the Fe LMM Auger peak, corroborating that Fe was an impurity. Finally, Ni-MO also showed the presence of Ni^2+^ and Ni^3+^ but with an increased Ni^2+^:Ni^3+^ ratio of 3.8 (previously 3.0).

The enhanced stability of the X-MO coatings was further confirmed by performing a more detrimental electrochemical test (5000 CVs, 100 mV s^–1^, totalling 24 h). The most noticeable difference was observed in Mn-MO where coating dissolution led to exposed minor parts of the FTO substrate (Figure S14a). This was also evidenced when evaluating the Mn L_2,3_-edges and the O K-edge (Figure S15) where an increase in contribution from Mn^3+^ was observed after the stability test. Noteworthy, Fe-MO, Co-MO, and Ni-MO mostly retained their initial characteristics except for the formation of tunnel-like structures and the opening of various spherical particles (Figure S14a). EDX elemental analysis reveals that the X:Sb (X = Mn, Fe, Co, or Ni) and Mo:Sb ratios closely resemble that of the pristine X-MO coatings (Figure S14b, Table S9) confirming the overall stability of the quaternary mixed-oxides. In particular, for Co-MO the NEXAFS Co L_2,3_-edge spectra do not show any energy shift or change in relative intensity after the stability test, indicating that no major changes in the valence state or spin configuration occurred (Figure S16a). Moreover, Co-MO also retains good stability after a 24 h CA test (Figure S16b,c).

### Activity and Stability under Alkaline and Anodic Potentials

**Figure 8:**
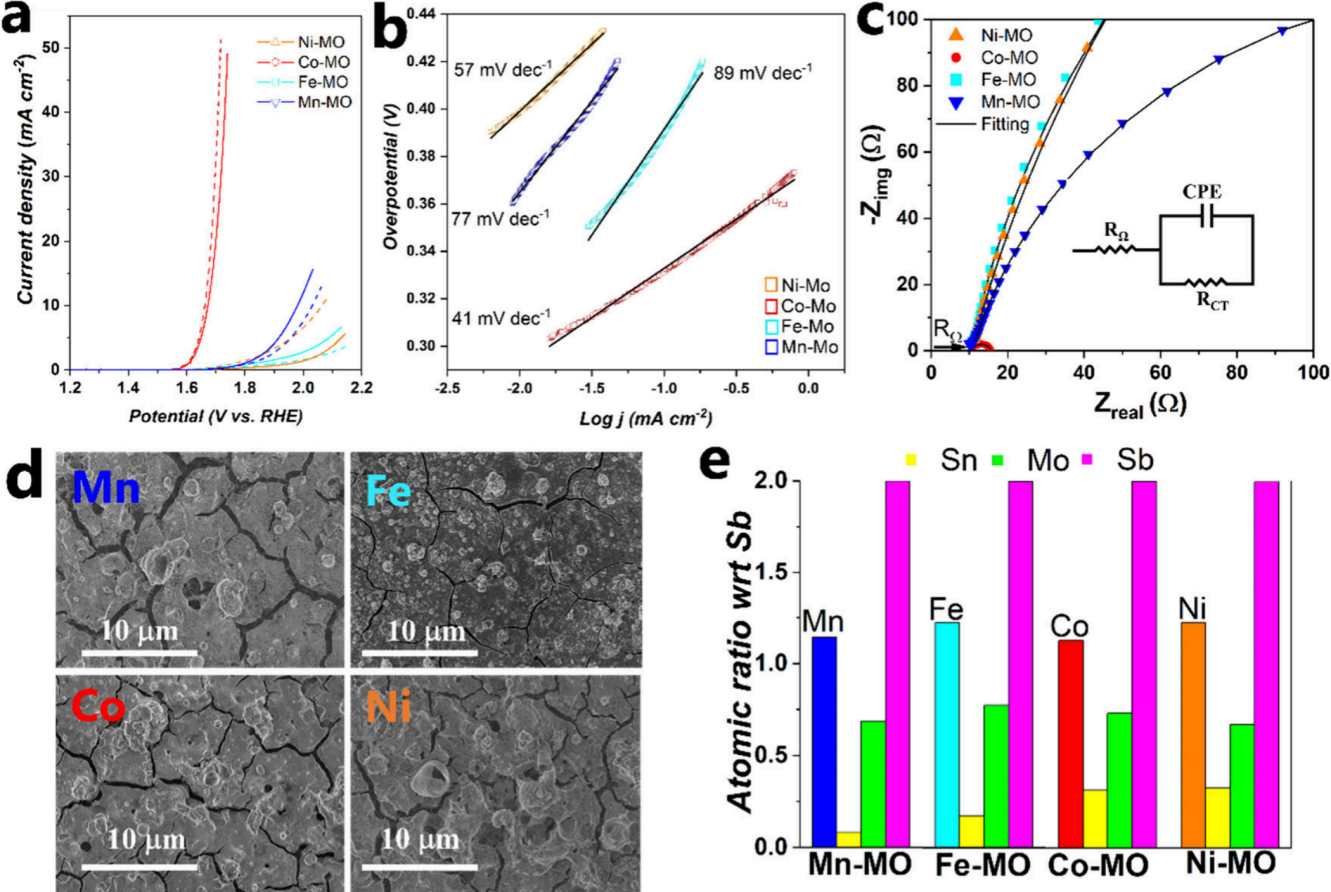
(a) *iR*-corrected polarization curves of X-MOs coatings prepared on FTO in 1 M KOH using 1 mV s^–1^ scan rate (dash lines corresponds to the activity after stability test). (b) Tafel slopes evaluated at a 1 mV s^–1^ scan rate in KOH = 1 M. (c) Electrochemical impedance spectroscopy of X-MOs in KOH = 1 M. Nyquist plot evaluated at 1.7 V vs RHE; the inset shows the equivalent circuit model. The values of *R*_Ω_ and *R*_CT_ are listed in Table S6. (d) SEM images. (e) Metal content normalized with respect to Sb. The atomic ratios are 1.14:0.08:0.68:2, 1.22:0.17:0.77:2, 1.12:0.31:0.73:2, and 1.12:0.32:0.67:2 for X:Sn:Mo:Sb, where X = Mn, Fe, Co, or Ni.

Naturally, we also evaluated the OER activity of the X-MO coatings, and the results are shown in Figure 8a. The trend observed to trigger the OER is similar the one seen in acid electrolyte, Co-MO being the most active electrocatalyst followed by Mn-MO, Fe-MO, and Ni-MO. Co-MO presented an overpotential at η_10_ and η_50_ of 440 and 520 mV, respectively, whereas Mn-MO had an overpotential at η_10_ of 750 mV. The Tafel slope (Figure 8b) has the following trend Fe-MO (89 mV dec^–1^) > Mn-MO (77 mV dec^–1^) > Ni-MO (57 mV dec^–1^) > Co-MO (41 mV dec^–1^). The latter indicates that the water adsorption to form hydroxyl species via one electron transfer is controlling the reaction, as depicted in eq 2:^69^
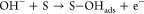
2However, Co-MO exhibited a Tafel slope of 41 mV dec^–1^, indicating that the reaction is limited by the formation of intermediary species with lower energy levels, as illustrated in eqs 3 and 4:^69^
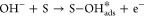
3
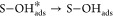
4

In eqs 2–4, S–OH_ads_^*^ and S–OH_ads_ denote intermediary species with different energy levels. The enhanced OER kinetics seen in Co-MO is also evidenced by EIS studies revealing a similar *R*_Ω_ and a lower *R*_CT_ when compared with those of the rest of the X-MO catalysts (see Table S6 and Figure 8c).

Samples sprayed on Ni-mesh (X-MO@Ni) demonstrated higher current densities, significantly lower overpotentials, and charge transfer resistances when compared to coatings prepared on FTO (Figure S17, Table S7). The performance observed for X-MO@Ni is comparable to other mixed oxides (Table S5). However, Tafel slopes were consistently higher, likely due to unaccounted side reactions at the Ni substrate, limiting the accurate evaluation of the Tafel slope. This is evident from the features observed at approximately 1.8 V vs RHE in the polarization curves (Figure S17a). The ECSA of X-MO@Ni coatings in alkaline conditions was 5–10 times greater than that observed in the acidic electrolyte. In alkaline conditions, ECSA values (expressed as capacitance of the double layer) ranged from 10.8 to 26.6 mF cm^–2^, with Co-MO@Ni demonstrating the smallest ECSA (Figure S18, Table S8) but the highest OER activity, once again suggesting that Co-MO possesses the highest specific activity among the X-MO coatings.

The stability of X-MO coatings was also assessed under an alkaline environment in the potential window 1.2–2.0 V vs RHE by applying a series of fast CVs (Figures S19 and S20) with a subsequent chronoamperometry test for 24 h (Figures S21 and S22). SEM studies and EDX elemental analysis, Figure 8d,e, reveal negligible variations in the morphology, elemental distribution, and atomic ratio after the electrochemical stress test when compared to their pristine counterparts; see also Table S10. These results confirm the excellent stability of the X-MOs under alkaline conditions and anodic potentials. It is noteworthy to mention that once again, Mo corrosion was avoided even when its dissolution occurs in the entire anodic potential range studied here,^70^ indicating that its incorporation in the rutile-type structure reduce notably its corrosion.

## Conclusions

A new family of quaternary mixed-oxides containing X–Sn–Mo–Sb (X = Mn, Fe, Co, or Ni) were produced via solution precursor plasma spraying onto various substrates. The mixed oxides formed a single-phase oxide with rutile crystal structure characterized by a random atomic motif within the crystal lattice. Due to the nature of the production process, we identified that the presence of an oxidizing agents such as (MoO_4_^2–^) or (WO_4_^2–^) was a key factor in the formation of the rutile crystal phase, likely promoting the conversion of Sb^3+^ to Sb^5+^ facilitating the octahedral coordination needed in the rutile structure. We showed that the incorporation of X–Sn–Mo–Sb into a rutile-phase enhances the stability of its components even after prolonged electrochemical stress, irrespective of the harsh environment. This feature was notorious for Mn, Fe, Co, and Mo which are known to be easily corroded under the typical conditions required to trigger the OER. The enhanced stability of the produced mixed oxides also highlights their potential applications as scaffolds to host and protect OER active metals. In addition, we identified that Co-MO and Mn-MO have promising OER activity in both acidic and alkaline electrolytes. NEXAFS spectroscopy revealed that in Co-MO there is an increase in electron occupancy in Co d-orbitals and the presence of high spin Co^2+^ (*O_h_*). We anticipate that additional optimization, such as incorporating noble metals, engineering defects, or increasing the surface area, might offer opportunities to further enhance the catalytic performance of these quaternary mixed oxides.

## Data Availability

The data generated in this study are available from the corresponding author upon reasonable request.

## Supporting Information Available

Structural parameters of Co-MO calculated from DFT, electrochemical activity, and stability data of additional samples, EDX elemental composition, and Raman and NEXAFS spectra of materials after stability test (PDF)
