# Supplementary material for: Quaternary Mixed Oxides of Non-Noble Metals with Enhanced Stability during the Oxygen Evolution Reaction
Source: ACS Appl Mater Interfaces. 2024 Oct 13;16(51):70429–41. doi: 10.1021/acsami.4c10234 (PMC11672481; doi:10.1021/acsami.4c10234)
Supplement: Supplementary file 1 — am4c10234_si_001.pdf [file am4c10234_si_001.pdf]

Supporting Information

Quaternary Mixed Oxides of Non-noble Metals with Enhanced Stability During the Oxygen Evolution Reaction

Alexis Piñeiro-García,[1, †] Xiuyu Wu,[1] Esdras J. Canto-Aguilar,[1] Alice Kuzhikandathil,[1] Mouna Rafei,[1] and Eduardo Gracia-Espino.[1,*]

[1] Department of Physics, Umeå University, SE-901 87 Umeå, Sweden.

[†] Current affiliation: Departamento de Ingeniería Química, Alimentos y Ambiental, Universidad de las Américas Puebla, Sta. Catarina Mártir, Cholula, Puebla 72810, Mexico.

[*]Corresponding author: Eduardo Gracia-Espino (eduardo.gracia@umu.se)

Table S1. *Comparison of the elemental composition of samples sprayed on FTO and Ti fibre felt. Metal ratio obtained from EDX normalized with respect to Sb.*

**Mixed oxide**	**Coating on FTO**<br>X:Sn:Mo:Sb	**Coating on Ti fibre felt**<br>X:Sn:Mo:Sb
Mn-MO	0.75:0.30:0.72:2	1.03:0.14:0.78:2
Fe-MO	1.19:0.34:0.82:2	1.16:0.31:0.89:2
Co-MO	0.92:0.19:0.87:2	0.99:0.23:0.92:2
Ni-MO	1.33:0.26:0.68:2	0.88:0.56:0.91:2

Table S2. *Variation in the relative peak intensity ratio and branching ratio of the NEXAFS spectra of Co* $L_{2,3}$*-edges.*

**Sample**	$I(L_3)/I(L_2)$	$I(L_3)/(I(L_3) + I(L_2))$
Co	1.44	0.59
Co-Sn-Sb	1.58	0.61
Co-Mo-Sb	2.34	0.70
Co-Sn-Mo-Sb	2.40	0.71
Co-Sn-W-Sb	2.47	0.72

Table S3. *Theoretical simulations to evaluate the feasibility of introducing Mo and Sn into rutile* $CoSb_2O_6$*. The system label indicates what atom Mo or Sn are replacing. Average value of the formation energy (*E_{form}*) relative to* $CoSb_2O_6$*, note that negative values indicate favourable structures. Average value of the geometrically optimized lattice parameters.*

**System**	**Description***	E_{form} **(eV) relative to** $CoSb_2O_6$**	***a*** **(Å)†**	***c*** **(Å)†**
$CoSb_2O_6$	1×1×1 trirutile unit cell. Two configurations tested.	0.00	4.7378	9.5036
Mo→ Co		-0.97	4.7898	9.7200
Mo → Sb		-1.45	4.7108	9.4586
Sn → Co		-0.48	4.8284	9.7102
Sn → Sb		-0.22	4.7162	9.4808
Sn → Sb	2×2×1 trirutile unit cell. 4 configurations tested.	-0.13	4.7177	9.5055
2Sn → 2Sb		-0.25	4.7160	9.5083
Mo → Sb<br>2Sn → 2Sb		-0.66	4.7201	9.4778
4Mo → 4Co<br>2Sn → 2Sb		-2.28	4.8418	9.8076
4Mo → 2Co + 2Sb<br>2Sn → 2Sb		-2.11	4.7773	9.6343

*The replaced atoms were randomly selected, and only constrained to the atomic species to be replaced.

**The formation energy was evaluated by using the equation $E_{form} = E_{tot} - \sum_i N_i \mu_i$, where E_{tot} is the total energy of the system, N_i and μ_i are the number of atoms and chemical potential of the species i. The systems used to evaluate the chemical potentials were hexagonal Co, bcc Mo, bcc Sb, bct Sn, and

molecular oxygen (O_2). The reported values are relative to a single trirutile of $CoSb_2O_6$ by simply setting its E_{form} to zero. The average value of all tested configurations is reported.

† The average value of all tested configurations is reported.

Table S4. *Theoretical simulations of a quaternary mixed oxide mimicking the Co-MO coating. The supercell was constructed using the rutile $CoSb_2O_6$ as starting point. The cation content was adjusted to emulate those seen experimentally.*

**Chemical element**	**Number of atoms***	**Atomic ratios**	**Atomic ratios from EDX and XPS experiments****
**System Co-MO-a**			
Co	42	Co/Sb = 0.51	Co/Sb = 0.50
Mo	34	Co/Mo = 1.23	Co/Mo = 1.13
Sn	10	Co/Sn = 4.2	Co/Sn = 4.16
Sb	82	Mo/Sb =0.41	Mo/Sb =0.44
O	383	Mo/Sn =3.4	Mo/Sn =3.67
		Sn/Sb =0.12	Sn/Sb =0.12
**System Co-MO-b**			
Co	40	Co/Sb = 0.48	Co/Sb = 0.50
Mo	36	Co/Mo = 1.11	Co/Mo = 1.13
Sn	8	Co/Sn = 5.00	Co/Sn = 4.16
Sb	84	Mo/Sb = 0.43	Mo/Sb = 0.44
O	384	Mo/Sn = 4.50	Mo/Sn = 3.67
		Sn/Sb = 0.10	Sn/Sb = 0.12

* The cell was constructed by replicating the *$CoSb_2O_6$* trirutile unit cell by 4×4×2 resulting in a nearly cubic supercell of ~19 Å in size (>550 atoms). The total number of cations was adjusted to maintain charge neutrality by considering their oxidation states found by XPS.

Data discussed in **Table 1 in the main manuscript.

Table S5. *Comparison of the performance towards the OER of mixed oxides in alkaline and acid media.*

Catalyst	Electrolyte	Molarity (M)	η_{10} (mV)	Tafel slope (mVdec^{-1})	Reference
**Mn-MO@Ni**	**KOH**	**1**	441	117	**This work**
**Fe-MO@Ni**	**KOH**	**1**	442	212	**This work**
**Co-MO@Ni**	**KOH**	**1**	425	99	**This work**
**Ni-CO@Ni**	**KOH**	**1**	497	227	**This work**
$Co_6Mo_6C_2$/ $Co_2Mo_3O_8$	KOH	1	403	87	[1]
$MnVO_x$	KOH	0.1	420	271	[2]
Fe_2O_3-MnO	KOH	1	370	66	[3]
$Co_3O_4/CoMoO_4$	KOH	1	318	63	[4]
Co–Cu–W-O_x	KOH	0.1	313	162	[5]
**Mn-MO@Ti**	H_2SO_4	1	$\eta_5 = 942$	153	**This work**
**Fe-MO@Ti**	H_2SO_4	1	$\eta_5 = 943$	185	**This work**
**Co-MO@Ti**	H_2SO_4	1	826	128	**This work**
**Ni-CO@Ti**	H_2SO_4	1	$\eta_5 = 934$	197	**This work**
Ni-Mn-Sb-O_x	H_2SO_4	1	735		[6]
$NiFePbO_x$	KP_i+KNO_3	pH= 2.5	-	90	[7]
IrO_2-α-MnO2	HClO4	0.1	317	74.7	[8]
RuO_2-TiO_2	H_2SO_4	0.5	180	43	[9]

Table S6. *Ohmic resistance (R_Ω) and charge transfer resistance (R_{CT}) of X-MO coatings prepared on FTO. EIS evaluated under acid and alkaline electrolytes.*

Mixed oxide	Acidic @ 2.2 V vs RHE ([H_2SO_4] = 0.5 M)		Alkaline @ 1.7 V vs RHE ([KOH] = 1M)	
	R_Ω (Ω)	R_{ct} (Ω)	R_Ω (Ω)	R_{ct} (Ω)
**Mn-MO**	9.9	16.6	10.7	281.9
**Fe-MO**	11.9	197.1	10.5	621.5
**Co-MO**	10.1	7.9	10.4	5.4
**Ni-MO**	10.9	76.9	10.5	913

Table S7. *Overpotential and Tafel slope for samples sprayed on Ti fibre felt, and Ni mesh.*

Mixed oxide	Titanium fibre felt (pH = 0)		Nickel mesh (pH = 14)	
	Overpotential (mV)	Tafel slope (mV dec^{-1})	η_{10} (mV)	Tafel slope (mV dec^{-1})
**Mn-MO**	$\eta_5 = 942$	153	441	117
**Fe-MO**	$\eta_5 = 943$	185	442	212
**Co-MO**	$\eta_{10} = 826$	128	425	99
**Ni-CO**	$\eta_5 = 934$	197	497	227

Table S8. *Capacitance of the double layer for samples produced on Ti fibre felt, and Ni-mesh.*

**Mixed oxide**	**Ti fibre felt. Evaluated at pH = 0 C_{dl} (mF cm^{-2})**	**Ni-mesh. Evaluated at pH = 14.0 C_{dl} (mF cm^{-2})**
**Mn-MO**	1.2	17.4
**Fe-MO**	1.9	26.6
**Co-MO**	2.0	10.8
**Ni-CO**	2.4	15.4

Table S9. *Elemental composition determined by EDX of X-MO coatings deposited on FTO as-produced, after 500 CVs (100 mV s^{-1}, 1.2 – 2 V vs RHE), and after 5000 CVs (100 mV s^{-1}, 1.2 – 2 V vs RHE) in acid electrolyte.*

**Mixed oxide**	**As-produced**	**After 500 CVs**	**After 5000 CVs**
**Mn:Sn:Mo:Sb**	**0.93**:0.33:0.93:2	**0.75**:0.30:0.72:2	**0.83**:1.16:0.4:2
**Fe:Sn:Mo:Sb**	**1.12**:0.25:0.91:2	**1.19:**0.34:0.82:2	**1.21**:0.19:0.71:2
**Co:Sn:Mo:Sb**	**1.01**:0.23:0.88:2	**0.92**:0.19:0.87:2	**0.97**:0.31:0.66:2
**Ni:Sn:Mo:Sb**	**1.17**:0.38:0.91:2	**1.33**:0.26:0.68:2	**1.03**:0.35:0.65:2

Table S10. *Elemental composition determined by EDX of X-MO coatings produced on FTO before and after stress test in alkaline electrolyte.*

**Mixed oxide**	**As-produced**	**After stress test***
**Mn:Sn:Mo:Sb**	**0.93**:0.33:0.93:2	**1.14**:0.08:0.68:2
**Fe:Sn:Mo:Sb**	**1.12**:0.25:0.91:2	**1.22**:0.17:0.77:2
**Co:Sn:Mo:Sb**	**1.01**:0.23:0.88:2	**1.12**:0.31:0.73:2
**Ni:Sn:Mo:Sb**	**1.17**:0.38:0.91:2	**1.12**:0.32:0.67:2

***** 1000 CVs (100 mV s^{-1}, 1.2 – 2 V vs RHE) followed by a chronopotentiometry test for 24 h at 10 mA cm^{-2} for Co-MO and Mn-MO, and 5 mA cm^{-2} for Fe-MO and Ni-MO.

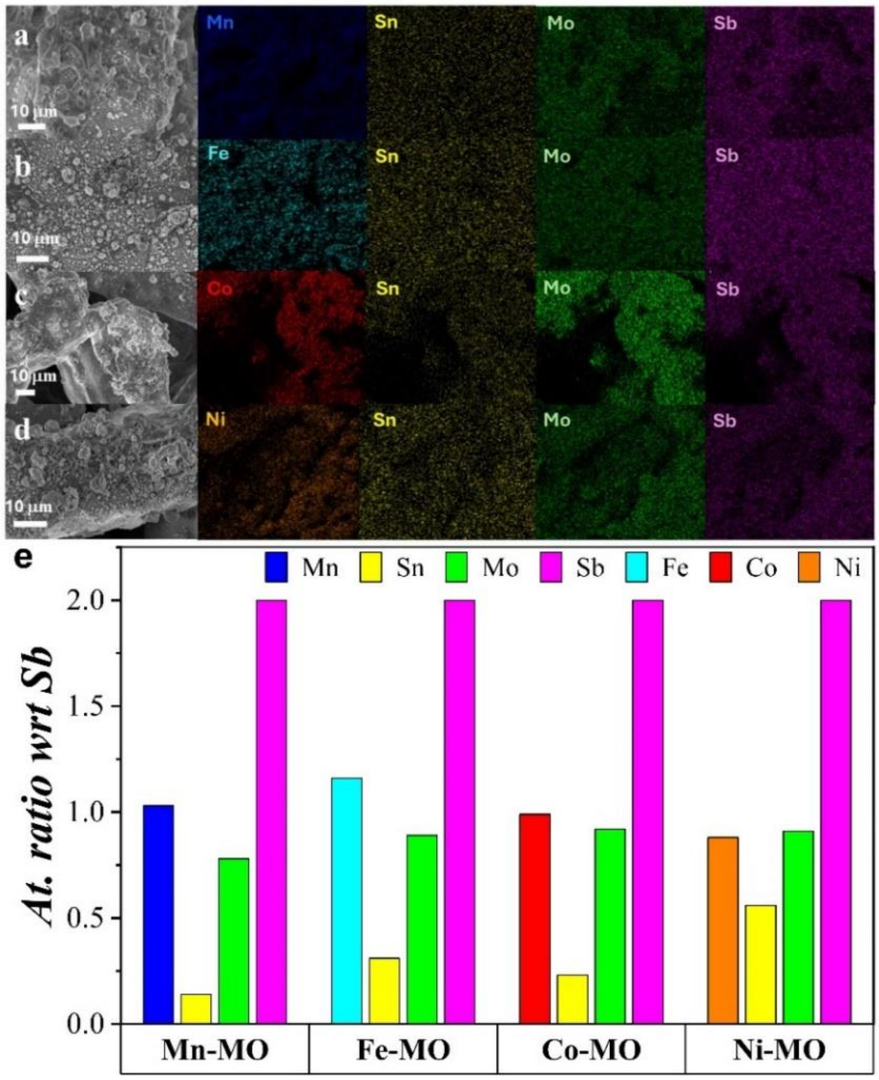

Figure S1. *Samples sprayed on Ti fibre felt. EDX elemental mapping of (a) Mn-MO, (b) Fe-MO, (c) Co-MO, and (d) Ni-MO. (e) Metal ratio obtained from EDX normalized with respect to Sb (Sb set to 2). The precursor solution had an initial metal ratio of 1:1:1:2 for X:Sn:Mo:Sb. The atomic ratios are 1.03:0.14:0.78:2, 1.16:0.31:0.89:2, 0.99:0.23:0.92:2, 0.88:0.56:0.91:2 for X:Sn:Mo:Sb, X = Mn, Fe, Co, or Ni.*

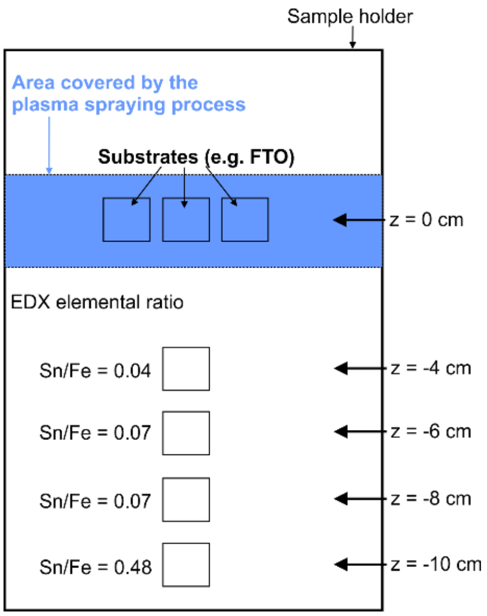

Figure S2. *Scheme of the sample holder and substrate position during the SPPS process. The blue rectangle marks the area where the plasma gun performs the raster scan during the coating process, in other words, where the coating is expected to form. The open black squares represent the positions where the substrates (e.g., FTO, Ti fibre felt, Ni-mesh) are placed. Those inside the blue area correspond to samples discussed in the manuscript. Samples outside the blue area were only used to investigate the reduce Sn content. The EDX elemental ratio of Sn/Fe is show.*

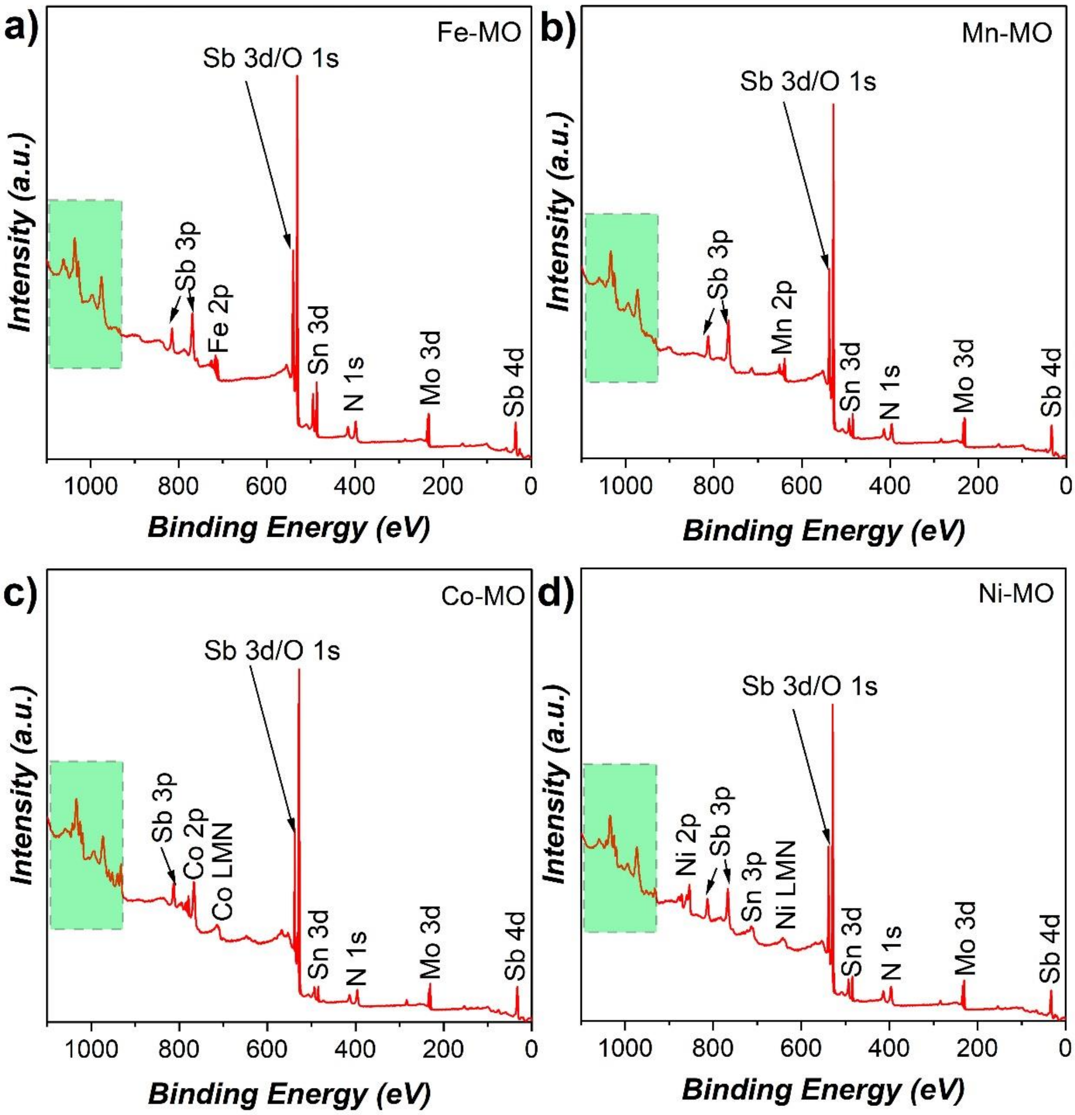

***Figure S3**. XPS survey spectra of X-MO coatings on FTO. (a) Fe-MO, (b) Mn-MO, (c) Co-MO, and (d) Ni-MO. The green rectangle indicates the Auger peaks from Sb MNN (>1000 eV), and O KLL (~970 eV). All samples exhibit N 1s features.*

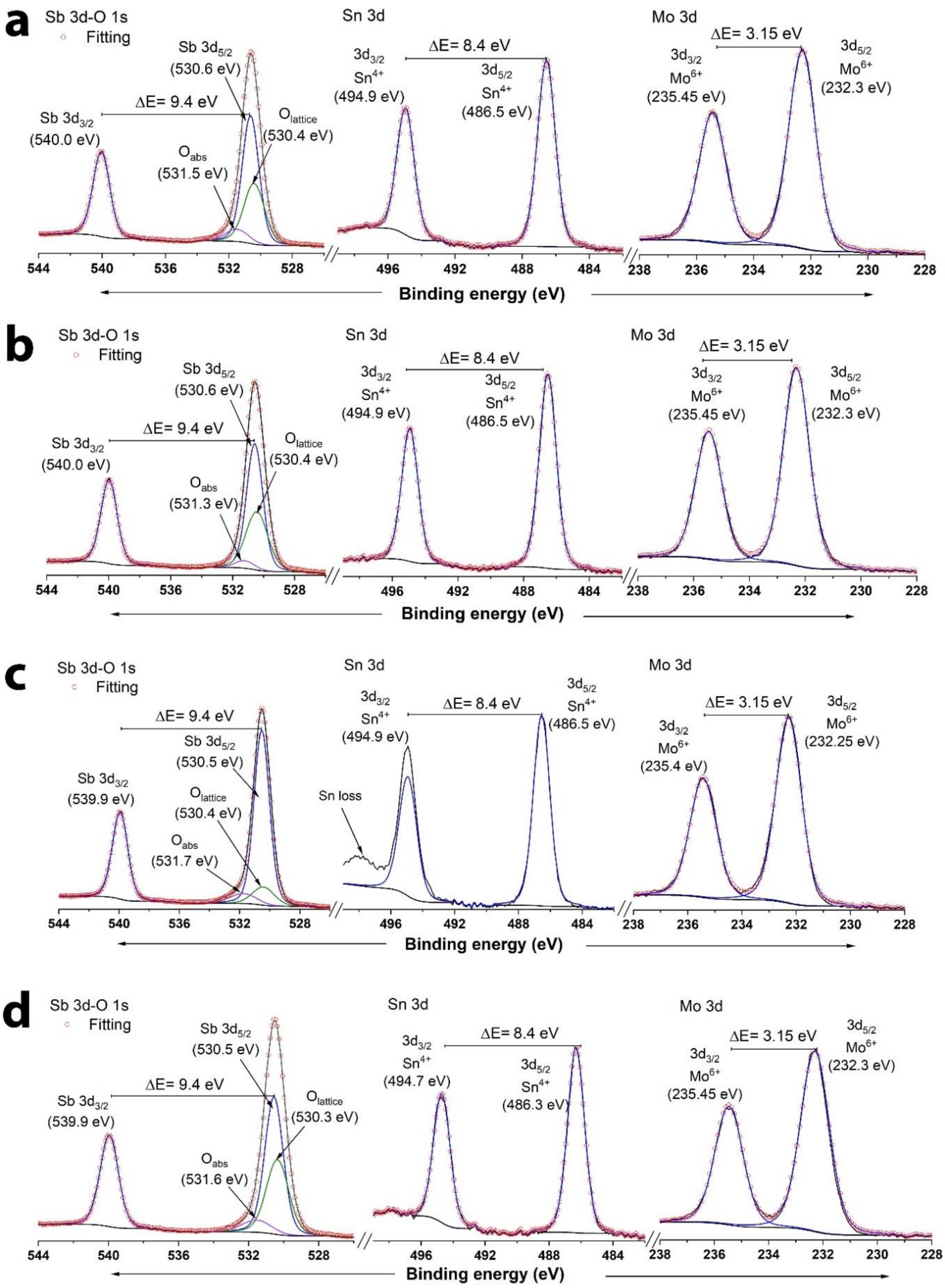

Figure S4. *High resolution XPS of X-MO coatings on FTO in the regions Mo 3d, Sn 3d, and Sb 3d/O 1s (right to left). (a) Mn-MO, (b) Fe-MO, (c) Co-MO, and (d) Ni-MO.*

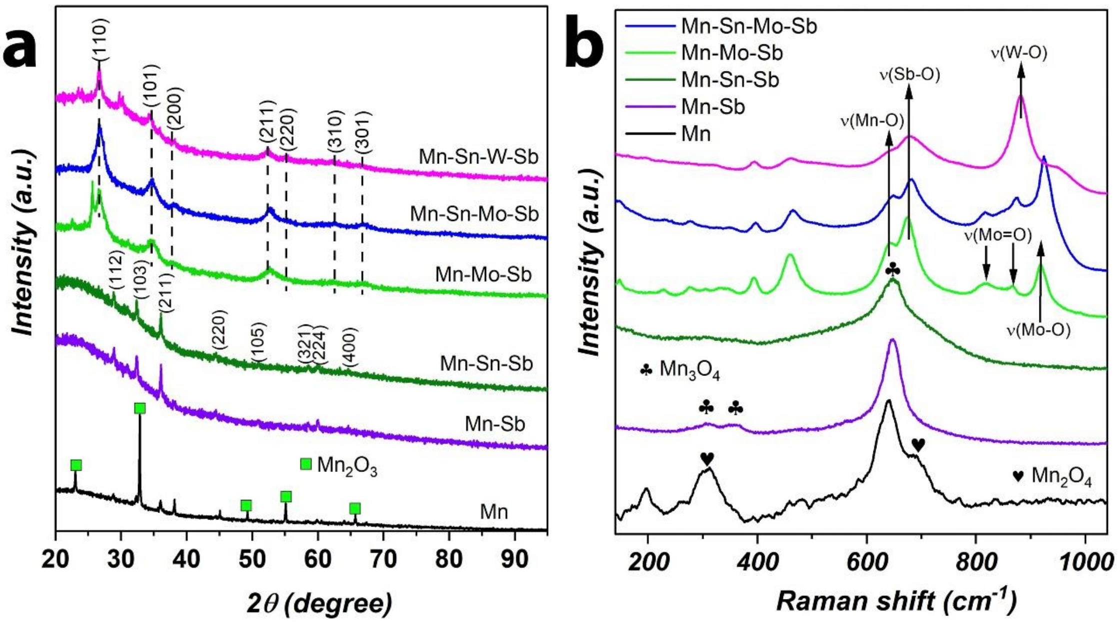

Figure S5. *Evolution of the crystal structure of Mn-MO produced on FTO. (a) XRD patterns, and (b) Raman spectra of oxides with different metal content. The samples with composition Mn, Mn-Sb and Mn-Sn-Sb revealed the formation of mainly* Mn_3O_4 *spinel structure, identified for the crystal planes (112), (103) and (211) and the Raman active modes at 648 cm*$^{-1}$*, 362 cm*$^{-1}$ *and 305 cm*$^{-1}$*. However, the single-rutile phase is formed upon addition of Mo or W-peroxo complexes, confirming that an oxidizing agent is needed to change the valence of* Sb^{3+} *to* Sb^{5+}*, a key feature required to construct the mixed oxides with single rutile-phase.*

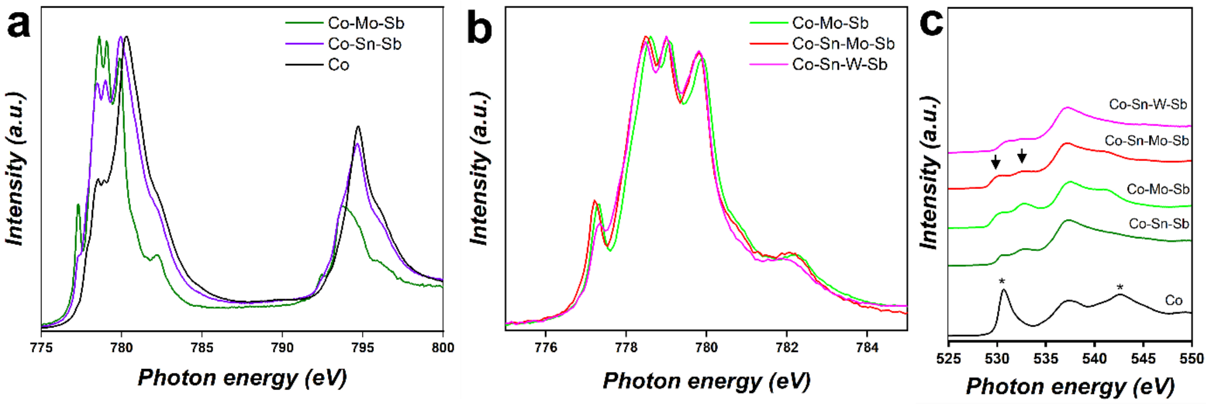

Figure S6. *X-ray absorption spectra of Co* $L_{2,3}$*-edges and O K-edge. Samples deposited on FTO. (a) Co* $L_{2,3}$*-edges for Co, Co-Sn-Sb, and Co-Mo-Sb. (b) Co* $L_{2,3}$*-edges for Co-Mo-Sb, Co-Sn-Mo-Sb, and Co-Sn-W-Sb. (c) O K-edge for Co, Co-Sn-Sb, Co-Mo-Sb, Co-Sn-Mo-Sb, and Co-Sn-W-Sb. Peaks in (c) marked with * correspond to* Co_3O_4*. Peaks marked with arrows agree with the presence of high spin* Co^{2+} *and the presence of 4d metals.*

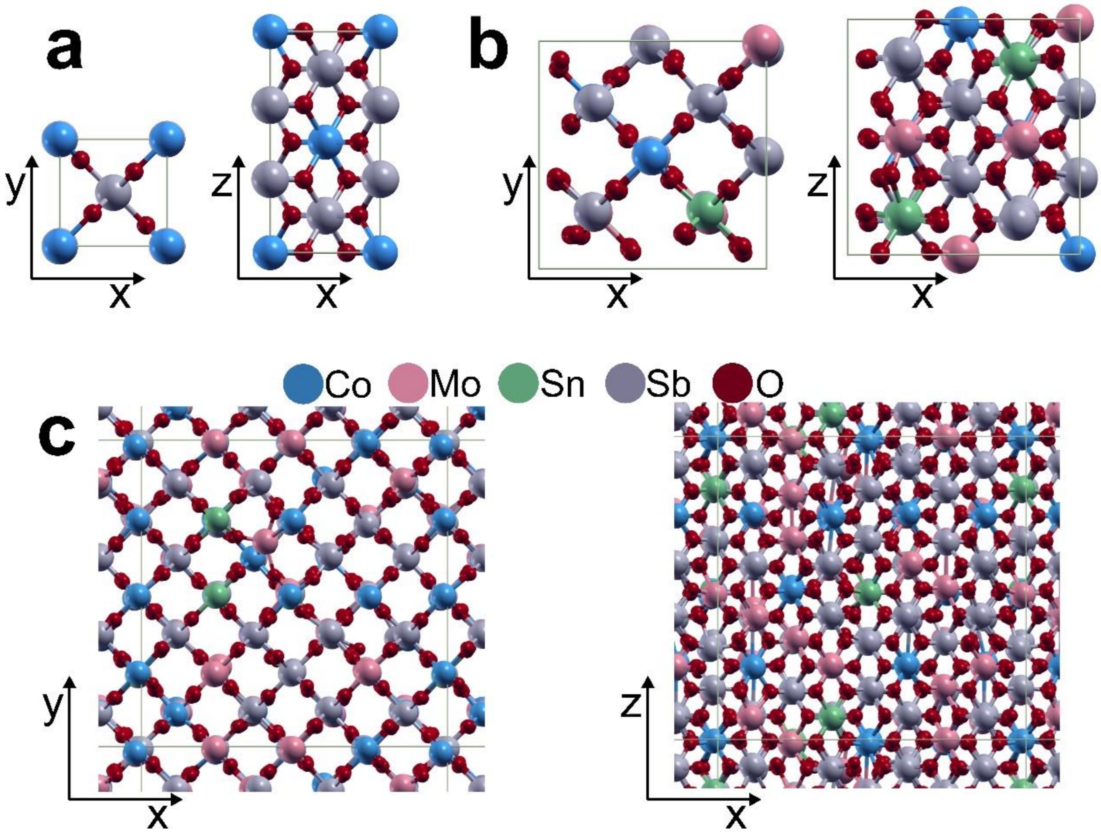

Figure S7. *Geometrically optimized atomic models. (a) Trirutile unit cell of* $CoSb_2O_6$*. (b) 2×2×1 cell used to evaluate the feasibility of introducing Mo and Sn. (c) Quaternary mixed oxide Co-MO-b. Elemental composition based on EDX and XPS results (Table 1). The cation occupation in the lattice was randomly assigned.*

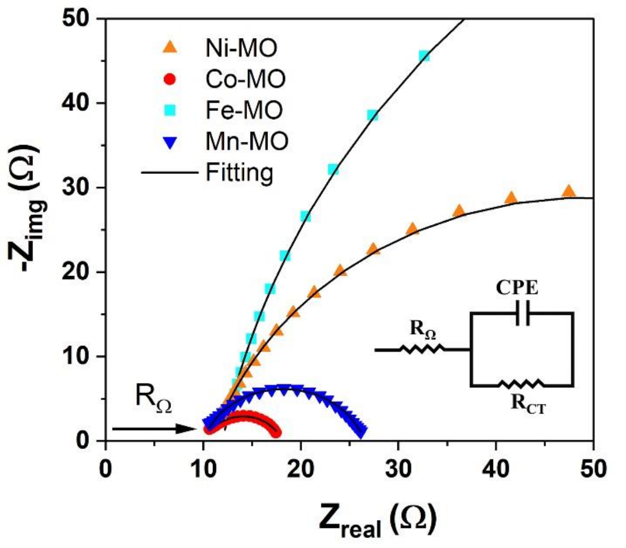

Figure S8. *Electrochemical impedance spectroscopy of X-MOs produced on FTO at pH=0. Nyquist plot evaluated at 2.2 V vs RHE; the inset shows the equivalent circuit model. The values of* R_{Ω} *and* R_{CT} *are listed in* ***Table S5***.

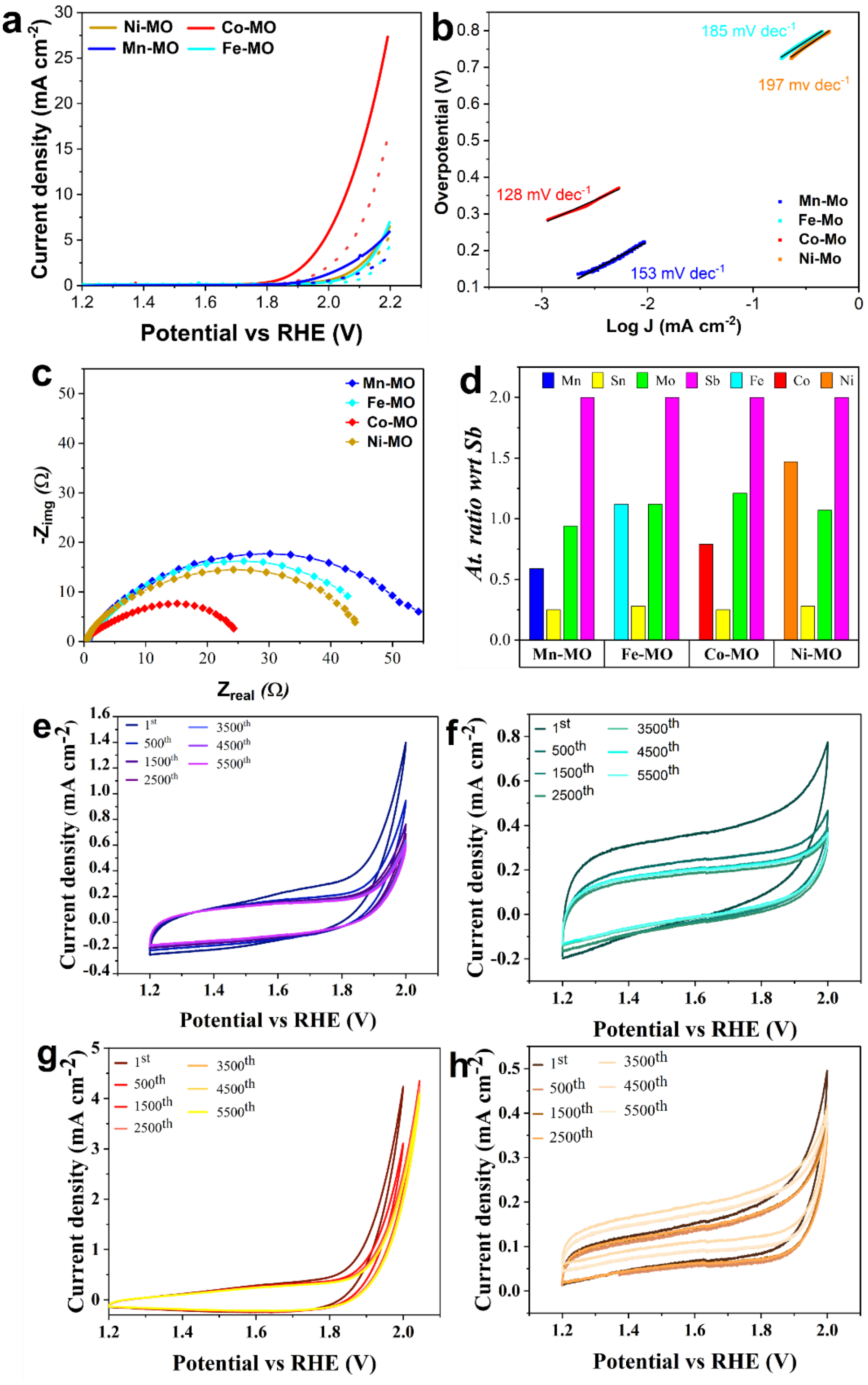

***Figure S9**. (a) iR-corrected polarisation curves, and (b)Tafel slope of X-MO coatings on Ti fibre felt. The OER activity was measured in 0.5 M H_2SO_4 with a scan rate of 5 mV s^{-1} (dash lines corresponds to the activity after 5500 CVs). (c) Nyquist plot obtained at 2.0 V vs RHE. (d) Metal ratio obtained from EDX normalized with respect to Sb (Sb set to 2). The precursor solution had an initial metal ratio of 1:1:1:2 for X:Sn:Mo:Sb. The atomic ratios are 0.59:0.25:0.94:2, 1.12:0.28:1.12:2, 0.79:0.25:1.21:2, 1.47:0.28:1.07:2 for X:Sn:Mo:Sb, X = Mn, Fe, Co, or Ni. (e-h) Stability test of X-MO coatings in acid electrolyte. Cyclic voltammetry test in the range 1.2 – 2.0 V vs RHE with a scan rate of 100 mV s^{-1}. A total of 5500 CVs were carried out at 100 mV s^{-1} (1.2 – 2.0 V vs RHE). (e) Mn-MO. (f) Fe-MO. (g) Co-MO. (h) Ni-MO.*

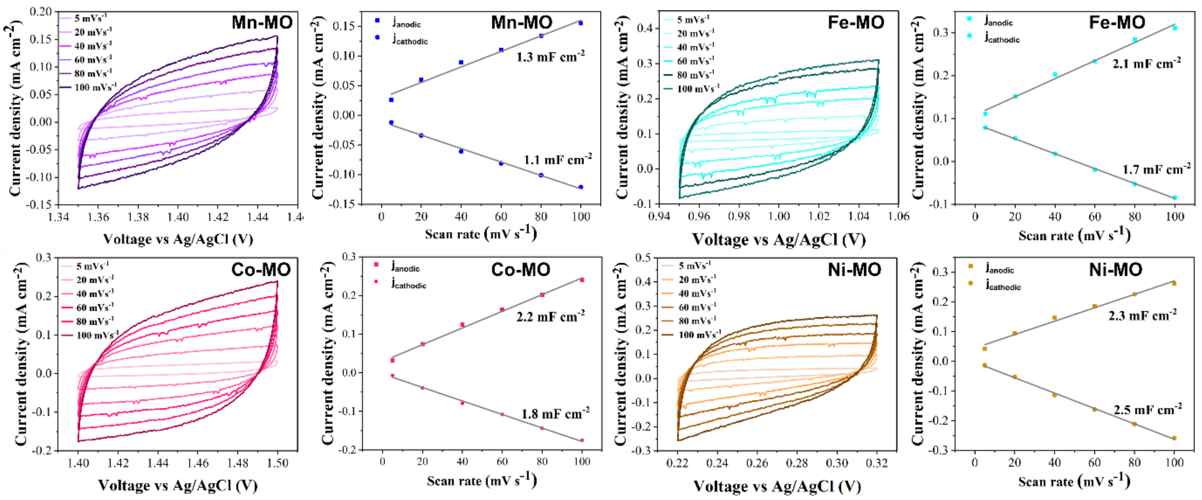

Figure S10. *Double layer capacitance measurement of samples produced on Ti fiber felt. Capacitance values are listed in* ***Table S7****.*

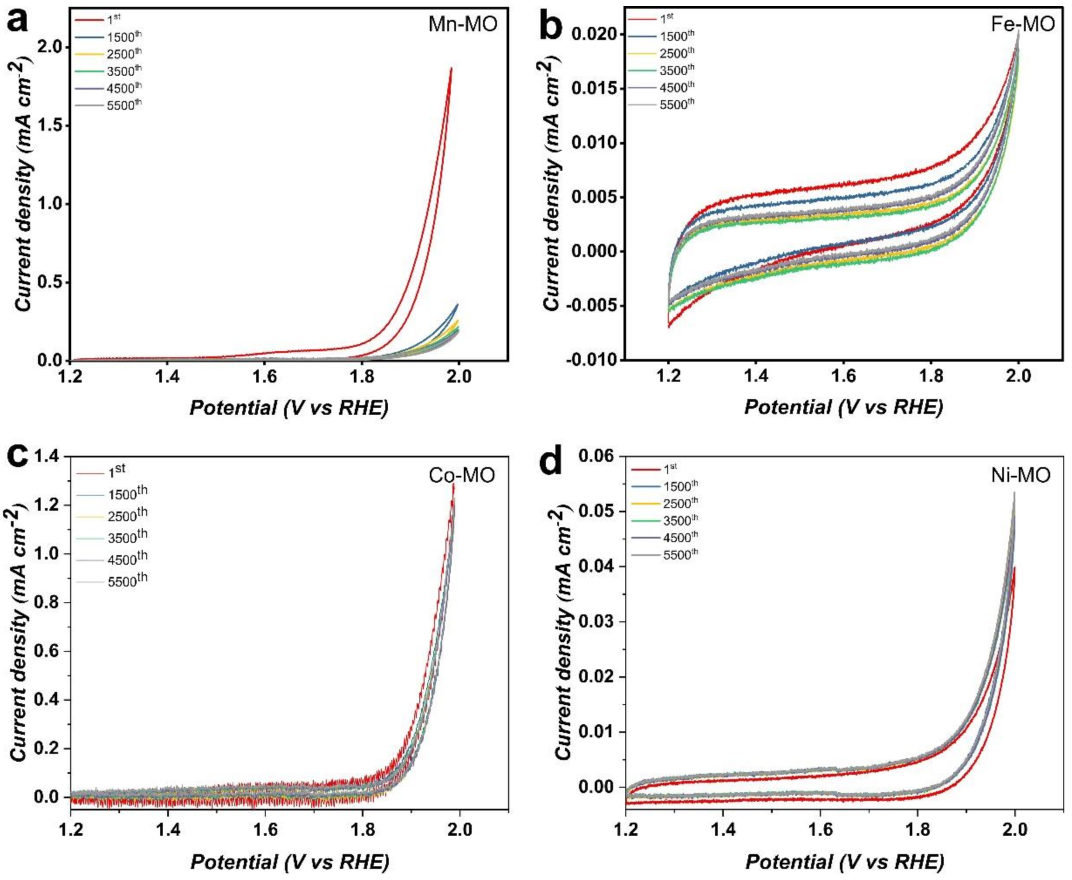

Figure S11*. Stability test of X-MO coatings on FTO in acid electrolyte. Cyclic voltammetry test in the range 1.2 – 2.0 V vs RHE with a scan rate of 100 mV* s^{-1}*. First a 500-CVs test was performed, and the samples were characterised. Subsequently a 5000-CVs test was carried out and samples were characterised once again. (a) Mn-MO. (b) Fe-MO. (c) Co-MO. (d) Ni-MO.*

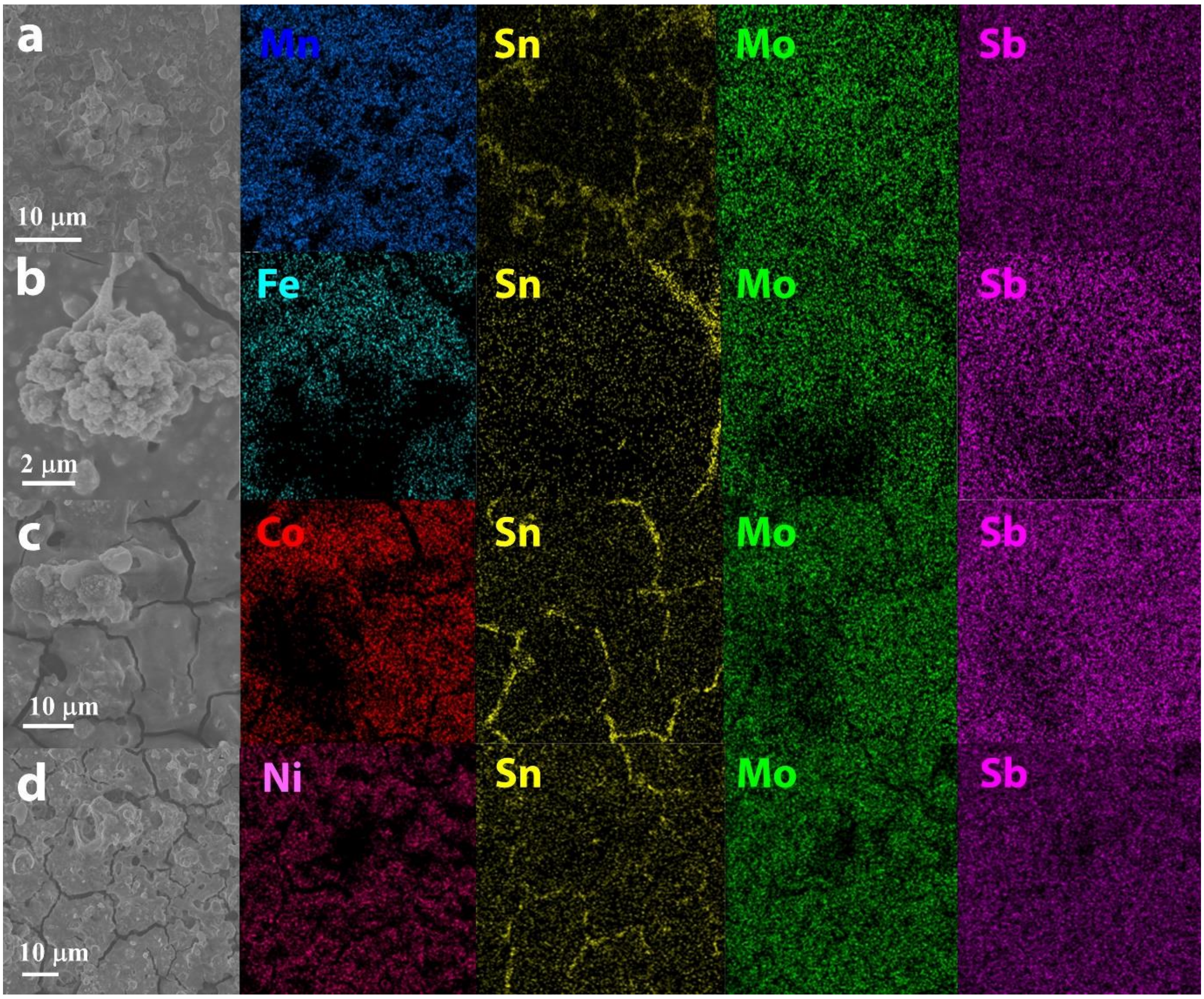

Figure S12. *EDX elemental mapping of X-MO coatings produced on FTO after 500 CVs at pH=0. (a) Mn-Sn-Mo-Sb, (b) Fe-Sn-Mo-Sb, (c) Co-Sn-Mo-Sb, and (d) Ni-Sn-Mo-Sb.*

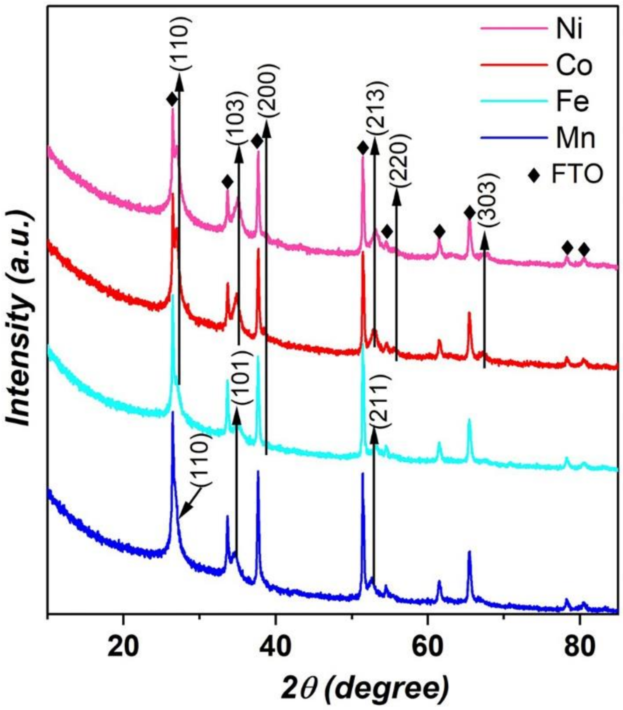

Figure S13. *XRD patterns of X-Sn-W-Sb (X = Mn, Fe, Co, Ni). XRD measurements performed on FTO substrate after 500 CVs at 100 mV s^{-1} in the potential range of 1.2 – 2 V in acid electrolyte. Note that the SnO_2 features observed by XRD in pristine Fe-MO (**Figure 3a**) are not detectable anymore, confirming that SnO_2 was not part of the mixed oxides.*

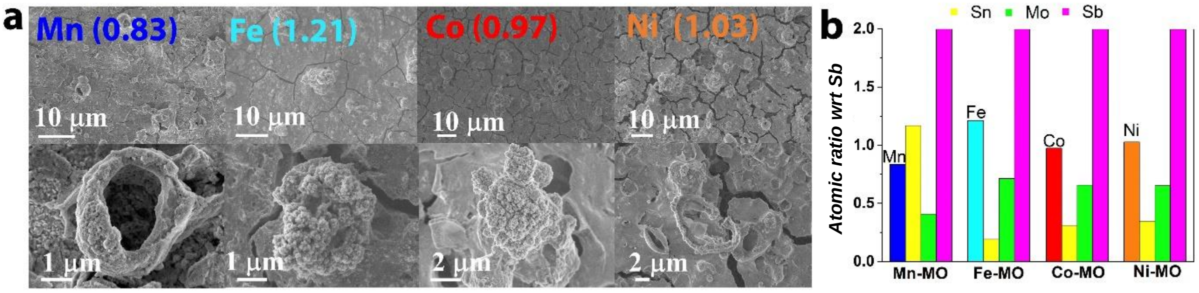

Figure S14. *(a) EDX elemental analysis of X-MO coatings produced on FTO after 5000 CVs at pH=0 and (b) Metal content normalized with respect to Sb. The atomic ratios are 0.83:1.16:0.4:2, 1.21:0.19:0.71:2, 0.97:0.31:0.66:2, and 1.03:0.35:0.65:2 for X:Sn:Mo:Sb, X = Mn, Fe, Co, or Ni. Note that Mn-MO exhibited a Sn:Sb of 1.16 due to partial exposure of the FTO substrate.*

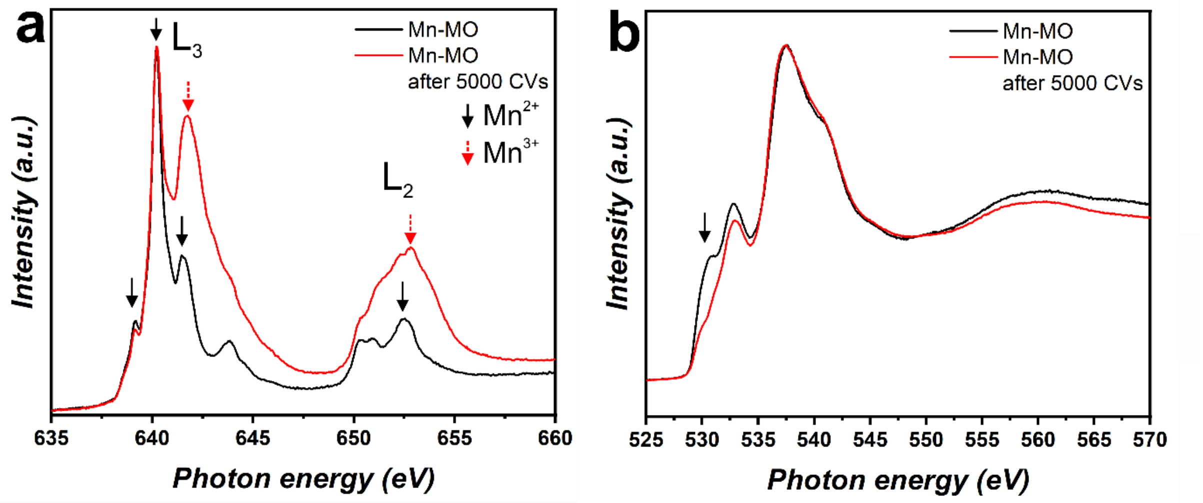

Figure S15. *X-ray absorption spectra before and after the stability test in acid electrolyte consisting in 5000 CVs at 100 mV* s^{-1}*, totalling 24 h. (a) Mn* $L_{2,3}$*-edges with characteristic features of both* Mn^{2+} *and* Mn^{3+} *that are octahedrally complexed by oxygen.* Mn^{3+} *characteristics are increased after stability test. (b) O K-edge with characteristic of coordination with both* Mn^{2+} *and* Mn^{3+}*. The decrease of the lower energy peak (marked by an arrow) is associated to a reduced contribution of high valence Mn, likely due to Mn dissolution during the stability test.*

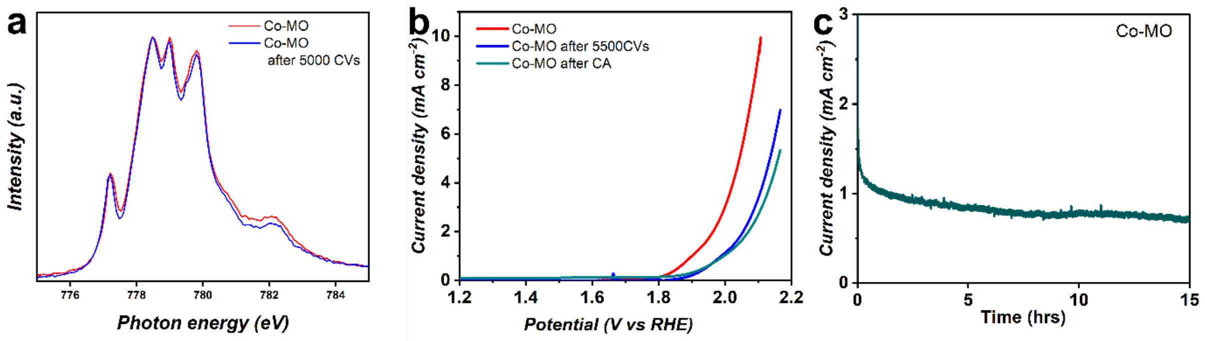

Figure S16. *Stability of Co-MO in acid electrolyte. (a) X-ray absorption spectra of Co* $L_{2,3}$*-edges before and after the stability test consisting in 5000 CVs at 100 mV* s^{-1}*, totalling 24 h. (b) Polarisation curves of Co-MO deposited on FTO as-produced, after 5000 CVs, and after 24h of CA. (c) CA test of the Co-MO sample shown in (b).*

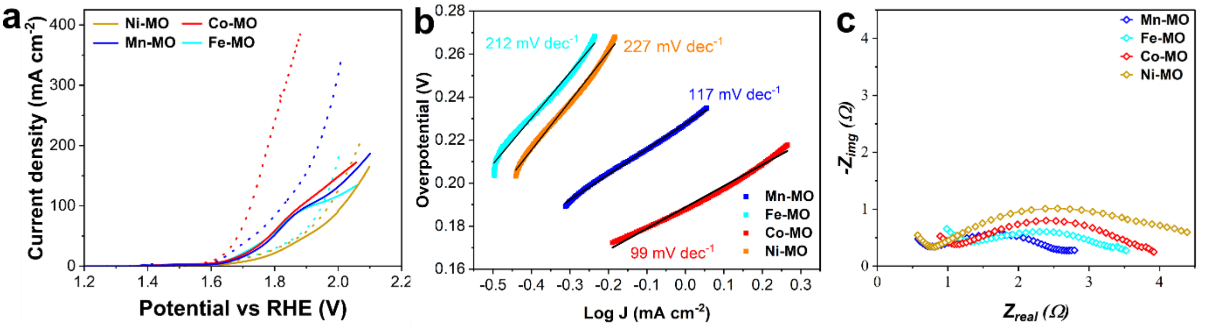

***Figure S17**. (a) iR-corrected polarisation curves of X-MOs coatings deposited on Ni-mesh in 1 M KOH using 5 mV s^{-1} scan rate (dash lines corresponds to the activity after stability test which correspond to 1000 CVs (1.2 – 2.0 V vs RHE, 100 mV s^{-1}) and 24 h CA (initial current density of 10 mA cm^{-2}). (c) Nyquist plot obtained at 1.8 V vs RHE.*

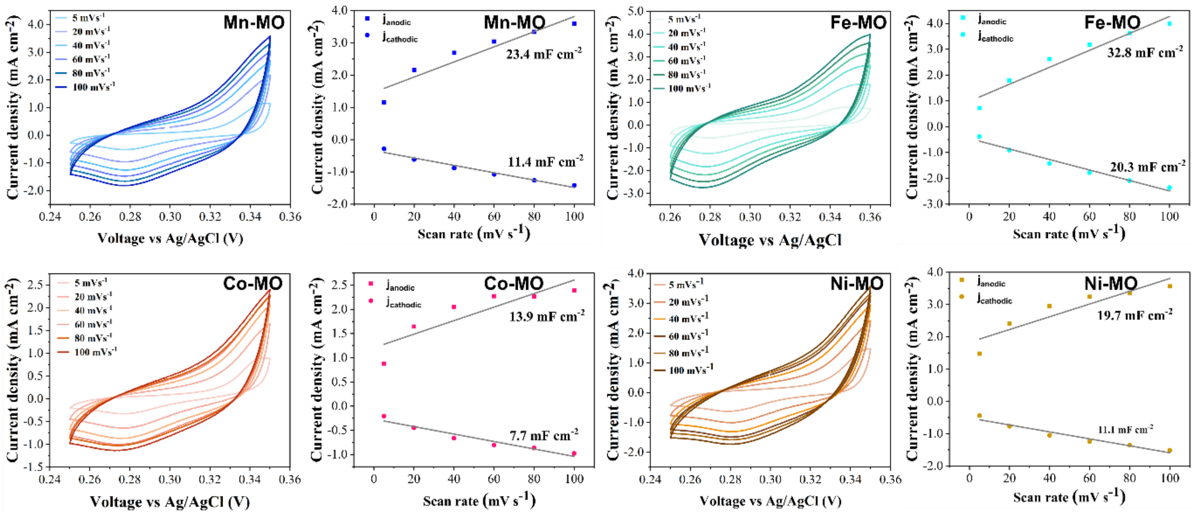

***Figure S18.** Double layer capacitance measurement of samples produced on Ni-mesh. Capacitance values are listed in **Table S7**.*

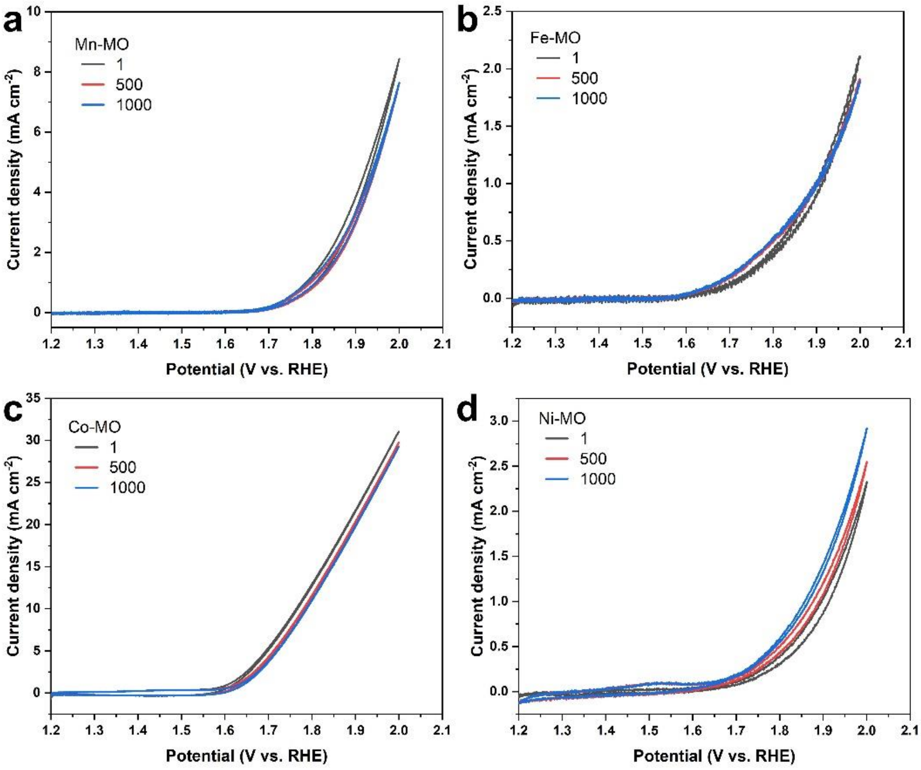

Figure S19. *Stability test of X-MO coatings deposited on FTO in alkaline electrolyte. Cyclic voltammetry test in the range 1.2 – 2.0 V vs RHE with a scan rate of 100 mV s^{-1}. (a) Mn-MO. (b) Fe-MO. (c) Co-MO. (d) Ni-MO. Only the CV cycle 1, 500, and 1000 are shown.*

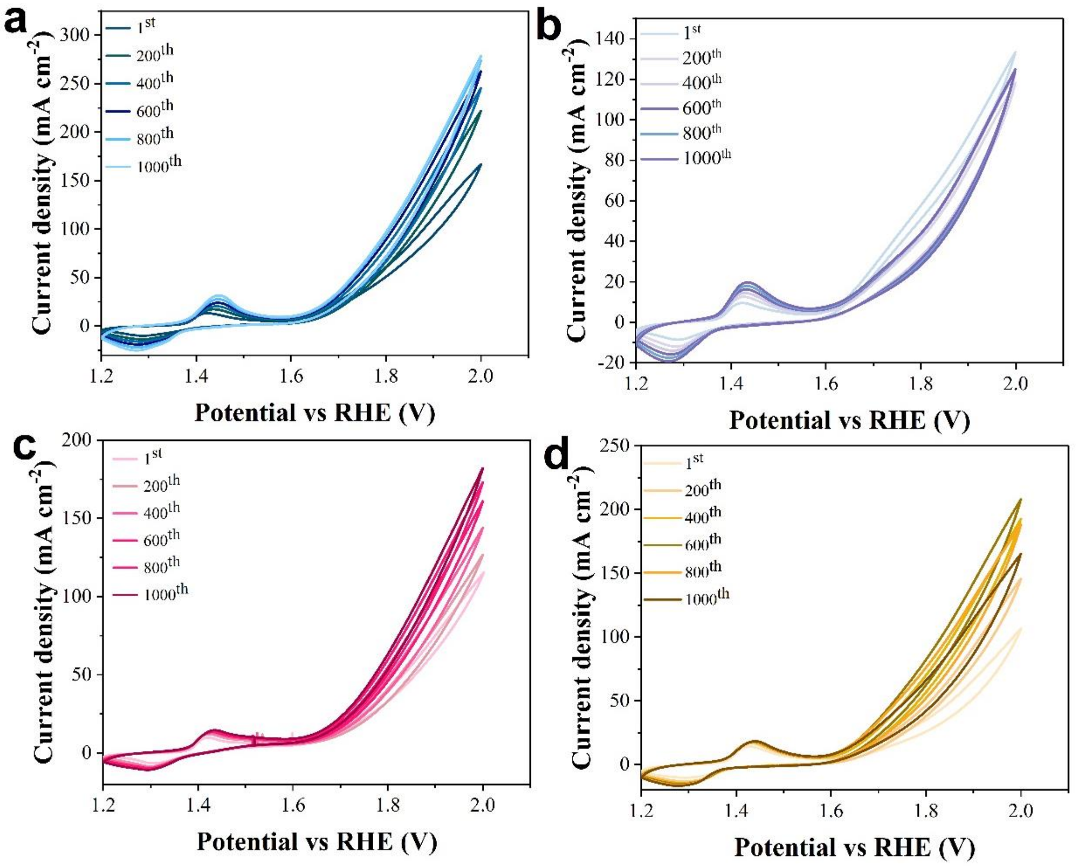

Figure S20. *Stability test of X-MO coatings deposited on Ni-mesh in alkaline electrolyte. Cyclic voltammetry test in the range 1.2 – 2.0 V vs RHE with a scan rate of 100 mV s^{-1}. (a) Mn-MO. (b) Fe-MO. (c) Co-MO. (d) Ni-MO.*

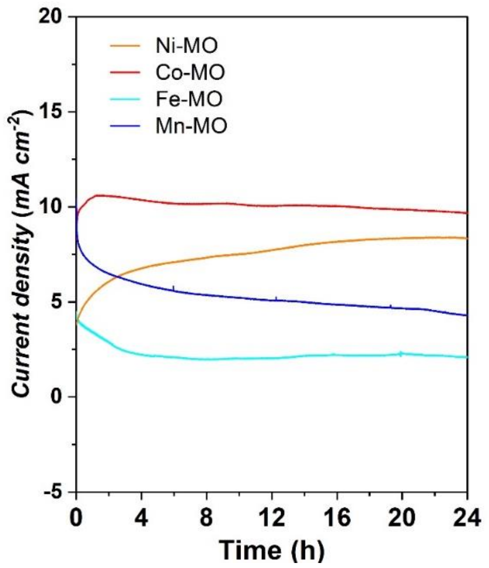

Figure S21. *Chronoamperometry test of X-MO coatings deposited on FTO. The CA tests was evaluated in alkaline conditions. The potential was set to achieve an initial current density of 10 mA cm^{-2} for Co-MO and Mn-MO. For Fe-MO and Ni-MO the potential was set to achieve an initial current density of 5 mA cm^{-2}.*

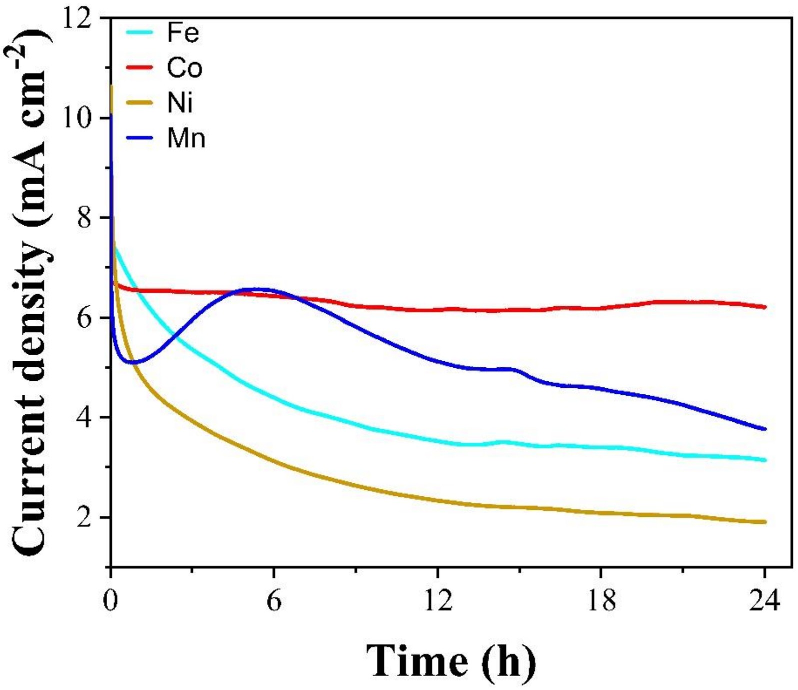

Figure S22. *Chronoamperometry test of X-MO coatings deposited on Ni-mesh. The CA tests was evaluated in alkaline conditions. The potential was set to achieve an initial current density of 10 mA cm^{-2} for all X-MO coatings.*

References

1. Liu, R., et al., *Bottom-up Design of Bimetallic Cobalt–Molybdenum Carbides/Oxides for Overall Water Splitting.* Chemistry – A European Journal, 2020. **26**(18): p. 4157-4164.
2. Xing, X., et al., *Manganese Vanadium Oxide–N-Doped Reduced Graphene Oxide Composites as Oxygen Reduction and Oxygen Evolution Electrocatalysts.* ACS Applied Materials & Interfaces, 2018. **10**(51): p. 44511-44517.
3. Kim, J., et al., *Electrochemical Synergies of Heterostructured Fe2O3-MnO Catalyst for Oxygen Evolution Reaction in Alkaline Water Splitting.* Nanomaterials, 2019. **9**(10): p. 1486.
4. Zhang, L., et al., *Hollow POM@MOF hybrid-derived porous Co3O4/CoMoO4 nanocages for enhanced electrocatalytic water oxidation.* Journal of Materials Chemistry A, 2018. **6**(4): p. 1639-1647.
5. Gupta, N., et al., *Catalytic Water Electrolysis by Co–Cu–W Mixed Metal Oxides: Insights from X-ray Absorption Spectroelectrochemistry.* ACS Applied Materials & Interfaces, 2024. **16**(27): p. 35793-35804.
6. Moreno-Hernandez, I.A., et al., *Crystalline nickel manganese antimonate as a stable water-oxidation catalyst in aqueous 1.0 M H2SO4.* Energy & Environmental Science, 2017. **10**(10): p. 2103-2108.
7. Li, N., et al., *Template-stabilized oxidic nickel oxygen evolution catalysts.* Proceedings of the National Academy of Sciences, 2020. **117**(28): p. 16187-16192.
8. Wang, Z., et al., *Influence of the MnO2 Phase on Oxygen Evolution Reaction Performance for Low-Loading Iridium Electrocatalysts.* ChemElectroChem, 2021. **8**(2): p. 418-424.
9. Wang, X., et al., *Electronic Structure Modulation of RuO2 by TiO2 Enriched with Oxygen Vacancies to Boost Acidic O2 Evolution.* ACS Catalysis, 2022. **12**(15): p. 9437-9445.
